# Direction of theoretical and experimental investigation into the mechanism of n-HA/Si-PA-SC@Ag as a bio-based heterogeneous catalyst in the reduction reactions

**DOI:** 10.1038/s41598-022-26200-3

**Published:** 2022-12-19

**Authors:** Zohreh Nouripour Sisakhti, Masoume Malmir, Masoumeh Bagheri Bisafar, Majid M. Heravi, Tayebeh Hosseinnejad

**Affiliations:** 1 Department of Organic Chemistry, Faculty of Chemistry, Alzahra University, Tehran, Iran; 2 Department of Physical Chemistry and Nano, Faculty of Chemistry, Alzahra University, Tehran, Iran

**Keywords:** Chemistry, Catalysis, Heterogeneous catalysis

## Abstract

In the present study, a natural-based heterogeneous catalyst is synthesized. For this purpose, nano-hydroxyapatite (n-HA) is prepared, silica-modified and functionalized with phthalimide. Finally, Ag^2+^ was immobilized onto n-HA/Si-PA-SC and reduced to Ag nanoparticles by *Bellis perennis* flowers extract. n-HA/Si-PA-SC@Ag characterized by TGA, FTIR, SEM/EDX, XRD, TEM, BET and ICP-AES techniques. Moreover, metal–ligand interactions in n-HA/Si-PA-SC@Ag complex models were assessed to make a quantitative representation for the immobilization behavior of Ag NPs on the surface of n-HA/Si-PA-SC through quantum chemistry computations. Furthermore, the performance of n-HA/Si-PA-SC@Ag was studied in the nitroarene, methylene blue and congo red reductions. Finally, the recyclability study as well as Ag-leaching verified that, n-HA/Si-PA-SC@Ag was stable and reused-up to four times without losing its activity.

## Introduction

Recently, an eco-friendly synthetic approaches have been one of the most important challenges for researchers. In this regard, catalytic processes are considered as a green method because they achieve specific chemical transformations in the shortest time in the presence of a small amount of catalyst and reduce production costs as well as environmental risks^1,2^ that natural and heterogeneous catalysts can be the best candidates^3^. In fact, nanomaterial-based catalysts have been extensively applied to accelerate processes while maintaining the principles of green chemistry^4,5^. In general, a heterogeneous catalyst with the unique characteristics of activity, stability and high selectivity is the developer of novel generation of solid-catalysts in the production of good chemicals for refinery operations and the environment^6^.

Nano-hydroxyapatite (n-HA) with Ca_10_(PO_4_)_6_(OH)_2_ molecular formula is one of the most usual types of calcium-phosphate. n-HA is an inorganic and heterogeneous nanomaterial with different properties depending on its preparation, such as the ability to form solid solutions and accept a large number of anionic and cationic substituents. Due to many properties of n-HA, such as high biocompatibility, low toxicity, absorption capacity, high surface-area, ion exchange capability and thermal stability, as well as similarity with the mineral phase of bone tissues, for many applications in industrial, medicine and catalytic reactions are known. Actually, n-HA has been a useful natural material as a new functionalized heterogeneous catalyst^6–15^. One of the important features of n-HA is structural flexibility that allows calcium or phosphorus ions to be replaced and its surface can to be functionalized^9–11^,^16^. In line with these issues, the remarkable catalytic activities of n-HA have been the ideal subject of numerous publications and patents, however, development of new methods for n-HA-based catalysts with good catalytic activities is needed^6,13,17^.

As an important class of active sites for promoting reactions, metal nanoparticles are versatile substrates in organic synthesis^15,18,19^. Meanwhile, among the greatest of the applications of silver nanoparticles (Ag NPs) is their use in the important and widely used reduction reaction, for which various metals are used. Ag NPs are a suitable option for these reactions due to their unique properties, especially their affordability and availability^15,19–23^.

Nitroarene compounds, which can be said to be relatively rare in nature, have entered the environment and caused pollution through human activities such as agriculture, dyeing, and some factories. Among them, nitrophenols are often known as toxic compounds of environment pollutants that they can easily affect life by contaminating sewage and food chain system^24,25^. In the environmental point of view, the synthesis of amines through the nitro-reduction process is one of the motivating reactions^15,26–28^. Organic reactions, especially the hydrogenation of nitro compounds, are very important in aquatic environments, and due to the lower-cost and ecological pollution, as well as higher-safety, they form the basis of some environmentally friendly research^15,29^.

On the other hand, dyes as key materials in textile, food, paper, food industries and pharmaceutical lead to environmental pollution, especially water wastage^30,31^. Therefore, the industrial effluent's control is essential to create a clean environment. Methylene blue (MB) as a cationic substance and Congo red (CR) as an anionic dye are extensively applied in industries for example, rubber, plastic and paper, which harm the environment if not cleaned in time^32^. Therefore, due to the importance and preservation of the environment, it is required to develop a simple manner for the efficient decomposition of dyes, which is expected to be achieved by Ag NPs because of their relatively large surface-to-volume ratio^33^.

In line with our investigations on the design, synthesis and computational modeling of heterogeneous catalysts and development of ecologically benign methods for chemical synthesis^34–39^ regarding the reports of researchers in the field of joining theory and experiments^40–42^, we have recently focused on the application of natural-heterogeneous catalysts in several organic conversions^19,43–45^. Hence, we are introducing efficient catalysts using n-HA decoration with organic functionalities and Ag-NPs doping by bio-assisted method. On the other hand, a quantitative description for metal–ligand interactions in n-HA/Si-PA-SC@Ag complex models is assessed by performing theoretical calculations using density functional theory to interpret the deposition of silver nanoparticles on the nano-rod hydroxyapatite support. Finally, it can be acknowledged that n-HA/Si-PA-SC@Ag has been used as a recoverable and heterogeneous nanocatalyst in the reduction reaction of nitroarene compounds as well as MB and CR with excellent yields.

## Result and discussion

### Catalyst characterization

After successful synthesis of the n-HA/Si-PA-SC@Ag, the catalyst structure was performed by several analyzes. It should be noted that the interpretation of FTIR, XRD, BET, TGA and SEM/EDX analyzes is described in SI.

**Figure 1:**
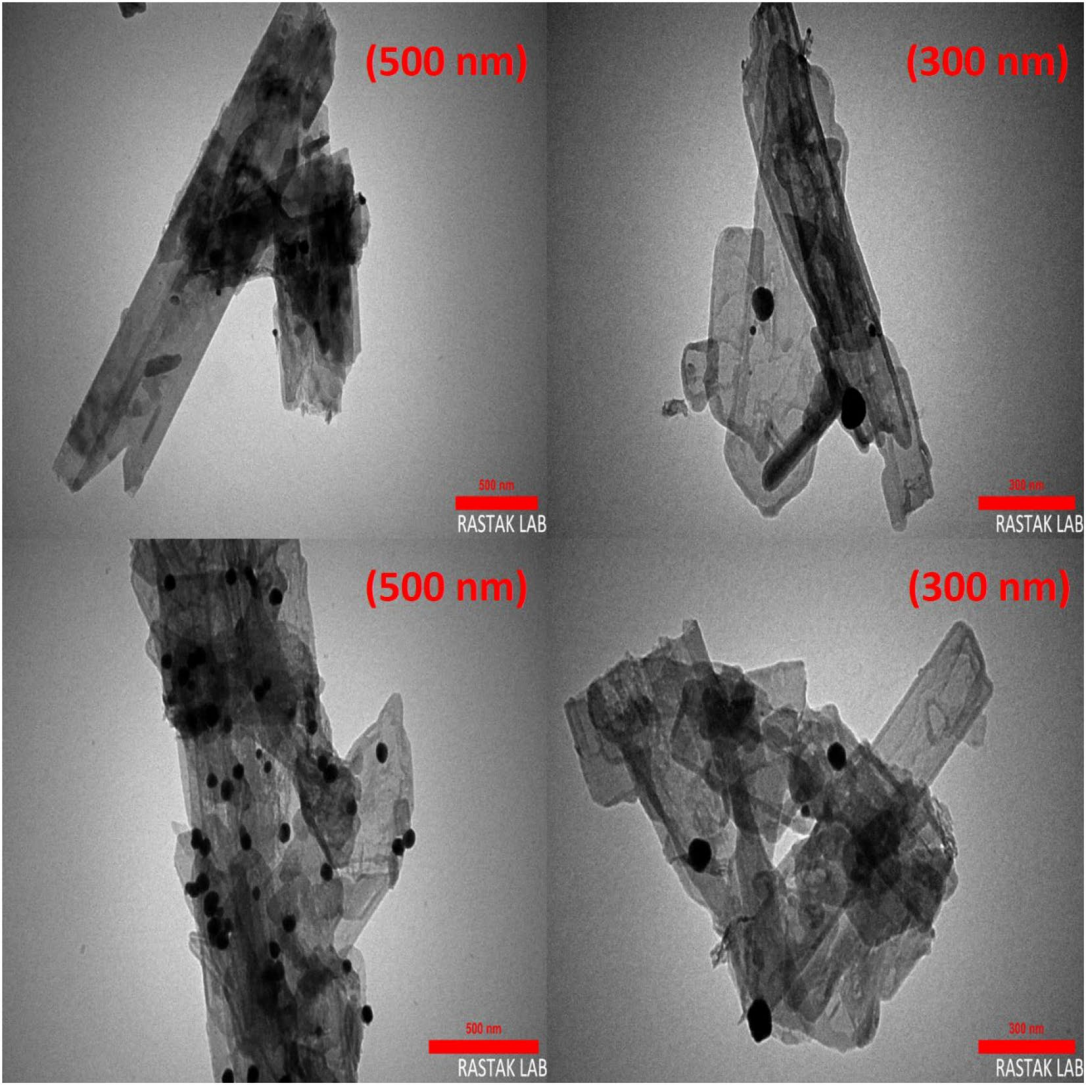
TEM images of n-HA/Si-PA-SC@Ag.

The morphological characteristics including the shape and particle size of n-HA/Si-PA-SC@Ag were investigated using TEM analysis (Fig. 1). According to the results obtained from SEM analysis, the nano-rod structure of n-HA is clearly visible. In addition, the uniformly distributed black dots on the surface of the n-HA/Si-PA-SC are a confirmation of the successful stabilization of Ag nanoparticles, which is consistent with the EDX results and XRD patterns. It should be noted that the average diameter of Ag nanoparticles is ~ 14 nm that is practically consistent with the result from the Debyee-Scherrer Eq. (10.9 nm). Finally, it can be acknowledged that this method has succeeded in effectively stabilizing Ag nanoparticles on the surface of n-HA/Si-PA-SC.

### Computational section

**Figure 2:**
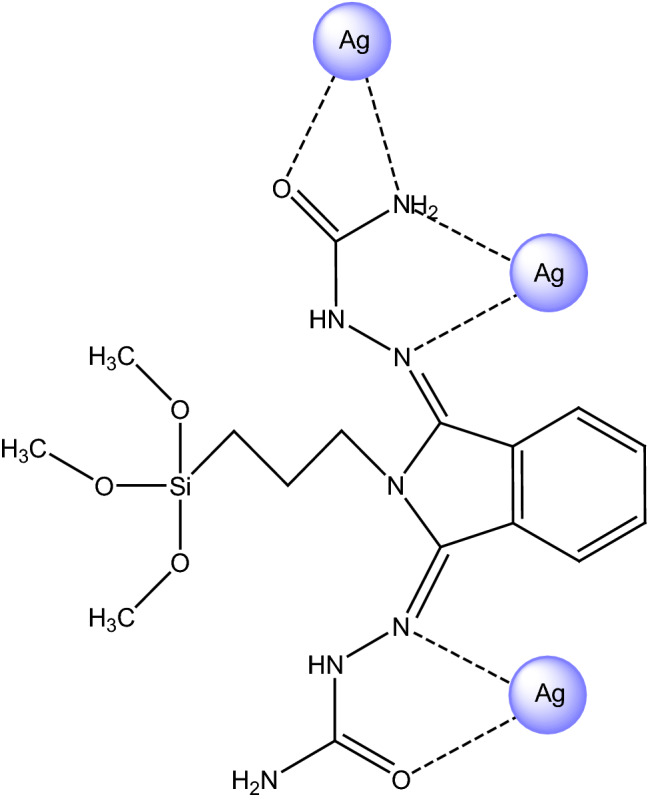
The designed structural model of n-HA/Si-PA-SC-Ag complex.

Recently, we have concentrated on the computational modeling of incorporation of transition metal nanoparticles (NPs) on a wide range of functionalized heterogeneous catalyst supports, in combination with the experimental assessments on their synthesis and applications in different organic reactions. In this line, we have investigated the immobilization behavior of palladium and copper NPs on the modified poly (styrene-co-maleic anhydride) surface. In addition, immobilization of Cu nanoparticles on the aminated and N-sulfamic-aminated KIT-5 nanocatalysts, poly(methyl methacrylate-co-maleimide) support and various functionalized halloysite nanoclays were modeled using quantum chemistry approaches^34,37,46–52^. In the recent year, we have assessed metal–ligand interactions in an appropriate designed model of n-hydroxyapatite supported-silver catalyst, functionalized with 4-aminoacetanilide^39^. In continuation of the above-mentioned joint experimental and computational researches, in this work, we have presented a reliable structural model of n-hydroxyapatite surface, functionalized with phthalimide and semicarbazide to investigate the complexation behavior of silver NPs in this heterogeneous nanocatalyst. It is significant to note that in order to present a computationally feasible approximation of large systems such as the surface of heterogeneous catalysts, periodic boundary conditions are often applied using unit cells as modeling boxes. During the computations only the properties of the original unit cell are calculated and propagated in the chosen dimensions. In this research, since we have investigated the immobilization behavior of AgNPs on the catalyst surface, we focused on the interaction of silver nanoparticles with phthalimide and semicarbazide segments of functionalized surface. So, we designed the complex models of Ag@n-HA@SiO_2_-PA-SC and performed non-periodic DFT computations, regardless of the whole surface of catalyst. As the first step, we have illustrated the proposed structural design of complex model (denoted as n-HA/Si-PA-SC-Ag) together with the possible coordination modes (Fig. 2). It should be noted that from the time and efficiency viewpoints in computational process, the suggested complex size has a reliable synchrony^36,53^. The optimized geometry of n-HA/Si-PA-SC ligand and n-HA/Si-PA-SC-Ag complex have been depicted in Fig. S6 which have been obtained through density functional theory (DFT) calculations at M06/6-311G** level^54^. Moreover, in Fig. S6, we have displayed the calculated values of bond order (together with the bond length in the parenthesis) for some key bonds in the coordination modes of n-HA/Si-PA-SC-Ag complex model. It is essential to emphasize that M06 functional has been classified as a highly parametrized exchange–correlation hybrid functional with meta-generalized gradient approximation that aims for a balanced description for both main-group and transition-metal chemistry. The performance of M06 functional has been assessed via a benchmarking calculation, comparing with 12 other functionals and Hartree–Fock theory for various databases, including thermochemistry, kinetics, noncovalent interactions, transition metal bonding, metal atom and molecular excitation energies, bond lengths, vibrational frequencies, and vibrational zero point energies. In order to verify the optimization procedure, we examined all real frequencies and all DFT computations have been performed using GAMESS suite of programs^55^. As it can be clearly extracted from Fig. S6 the calculated bond order values of selected N–N, C-O and C-N bonds have been fallen through metal–ligand interactions which can be directly due to the donation of shared electrons from this chemical bonds to silver atoms, which are obviously validated with our obtained FT-IR elucidations.

**Figure 3:**
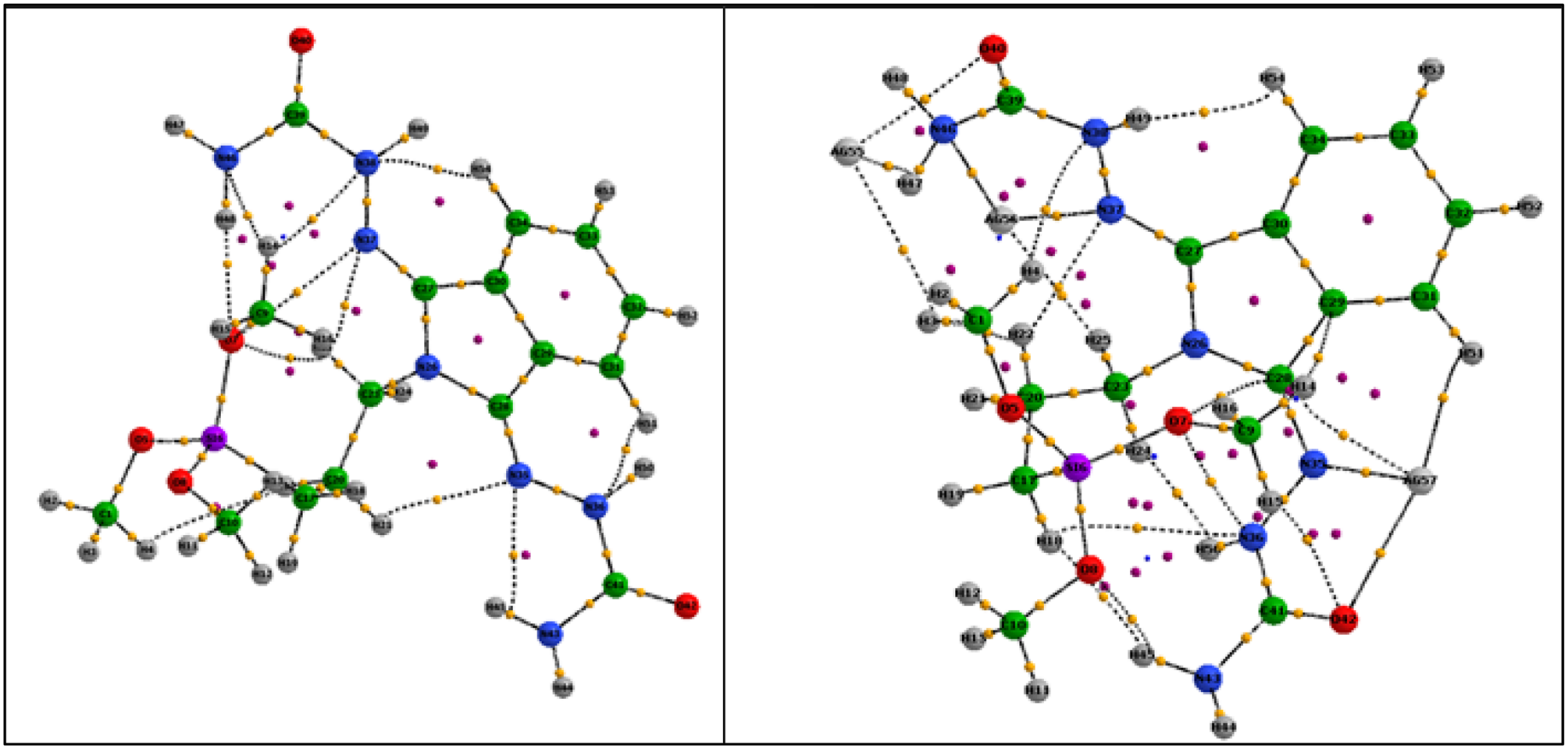
QTAIM graphs of n-HA/Si-PA-SC ligand and n-HA/Si-PA-SC-Ag complex model, obtained by the analysis of M06/6-311G** electron density functions. Bond Critical Points: yellow circles; Ring Critical Points: purple circles; Bond Paths: black lines.

In the next step, we employed the quantum theory of atoms in molecules (QTAIM) methods^56,57^ to analyze the topological properties of electron densities at the optimized structure of n-HA/Si-PA-SC ligand and n-HA/Si-PA-SC-Ag complex. To this end, we used the calculated M06/6-311G** wave function of the optimized geometry of n-HA/Si-PA-SC ligand and n-HA/Si-PA-SC-Ag complex as input files for AIM2000 program package^58^. In Fig. 3 we presented the QTAIM graphs of n-HA/Si-PA-SC ligand and n-HA/Si-PA-SC-Ag complex that demonstrate all bond and ring critical points and bond paths. Furthermore, we calculated the various QTAIM indicators such as electron density (ρb), its laplacian (∇^2^ρb), electronic kinetic energy density (Gb), electronic potential energy density (Vb), total electronic energy density (Hb) and the ratio of |Vb |/Gb which have been reported in Table S2, for some selected key bond critical points (BCPs) and ring critical points (RCPs) in n-HA/Si-PA-SC ligand and n-HA/Si-PA-SC-Ag complex.

The reported results of Table S2 reveal the reduction of electron density on N–N, C-O and C-N critical points via the coordination of these key chemical bonds to silver atoms, resulting in the descent of stretching frequency of IR spectrum. Moreover, the large values of electron density and the negative corresponding values of laplacian and H_b_ on the key C–C, C-N and C-O BCPs are in confirmation with the covalent nature of these bonds, whereas on Ag–N, Ag–O and Ag-H BCPs, the calculated ${\rho }_{b}$ values are smaller and ${\nabla }^{2}{\rho }_{b}$ values are positive, approving the semi electrostatic-covalent character of intramolecular bonding between silver atoms and ligand model. On the other hand, the calculated values of |Vb |⁄Gb for all Ag–N, Ag–O and Ag-H BCPs are between 1 and 2 that clearly affirm the partially electrostatic-covalent character of metal–ligand interactions in this model complex. Another significant aspect of QTAIM calculations can be concluded via the more precise analysis of QTAIM molecular graphs which portrays various intramolecular BCPs between silver atoms with nitrogen, oxygen, carbon and hydrogen atoms of n-HA/Si-PA-SC ligand that generates some new ring critical points (RCPs) and therefore, leading to the considerable electronic stabilization impact on the complexation procedure.

### Catalytic activity

Our goal in this project was the synthesis of a nanocatalyst that can effectively reduce nitro compounds and be cost-effective with a heterogeneous and recyclable nature. Therefore, first n-HA was prepared and after successful characterization, it was investigated in the reduction reaction of *p*-NP. For this purpose, First, the catalytic study of the n-HA in the reduction of *p*-NP (0.5 mmol) to its relevant *p*-AP over NaBH_4_ (7.5 mmol) and 30 mg of catalyst as a model reaction was preferred. Based on the obtained results, no amount of catalyst was able to promote and complete the reduction in the presence of NaBH_4_ amounts. As mentioned, our intention was to synthesize a recyclable catalyst, but after separating the bare n-HA catalyst from the reaction mixture, its semi-heterogeneous nature was revealed and more than half of it lost during the separation process. Therefore, to improve the catalytic activity and catalyst recovery process, the n-HA substrate was functionalized in several steps and in each stage its catalytic activity was investigated according to the above conditions. As expected, the use of SiO_2_ to aid heterogeneity was beneficial but had no effect on the reduction reaction process. Next, in order to understand the reason of what factors in the structure of the catalyst can play a role in the reduction process, the silver salt (AgNO_3_) that was available was used as a catalyst. Surprisingly, *p*-AP was obtained after 4 h with 70% yield but its separation and recovery were not sufficient for our purpose. In the following, organic and inorganic materials were used to functionalize the n-HA substrate and prepare the linker containing heteroatoms to load more silver metal. As tabulated, the functionalized n-HA substrates were incapable of reducing the nitro compound, but they created a strong interaction for Ag loading and preventing its leaching. Importantly, no products and no noticeable color changes were observed in the reactions of the metal-free catalysts (see Fig. S7 in SI.). It should be mentioned that the metal-free catalysts are not operative in reducing *p*-NP. This observation confirmed that *p*-NP reduction reaction was possible in the presence of Ag nanoparticles. By comparing the results of AgNO_3_ salt and n-HA/Si-PA-SC@Ag catalyst, it can be stated that AgNO_3_ salt are able to promote the reaction and its difference with n-HA/Si-PA-SC@Ag catalyst is in recovery and separation (Table S3, entries 12–16). Actually, n-HA/Si-PA-SC@Ag catalyst was easily separated by simple filtration from the reaction mixture and was recycled up to four times with high yields, while AgNO_3_ salt was difficult to separate and the investigation of recyclability was impossible. Based on these observations, it can be concluded that using the current protocol is effective in stabilizing Ag particles as an active site in the catalyst structure via bio-based pathway.

**Figure 4:**
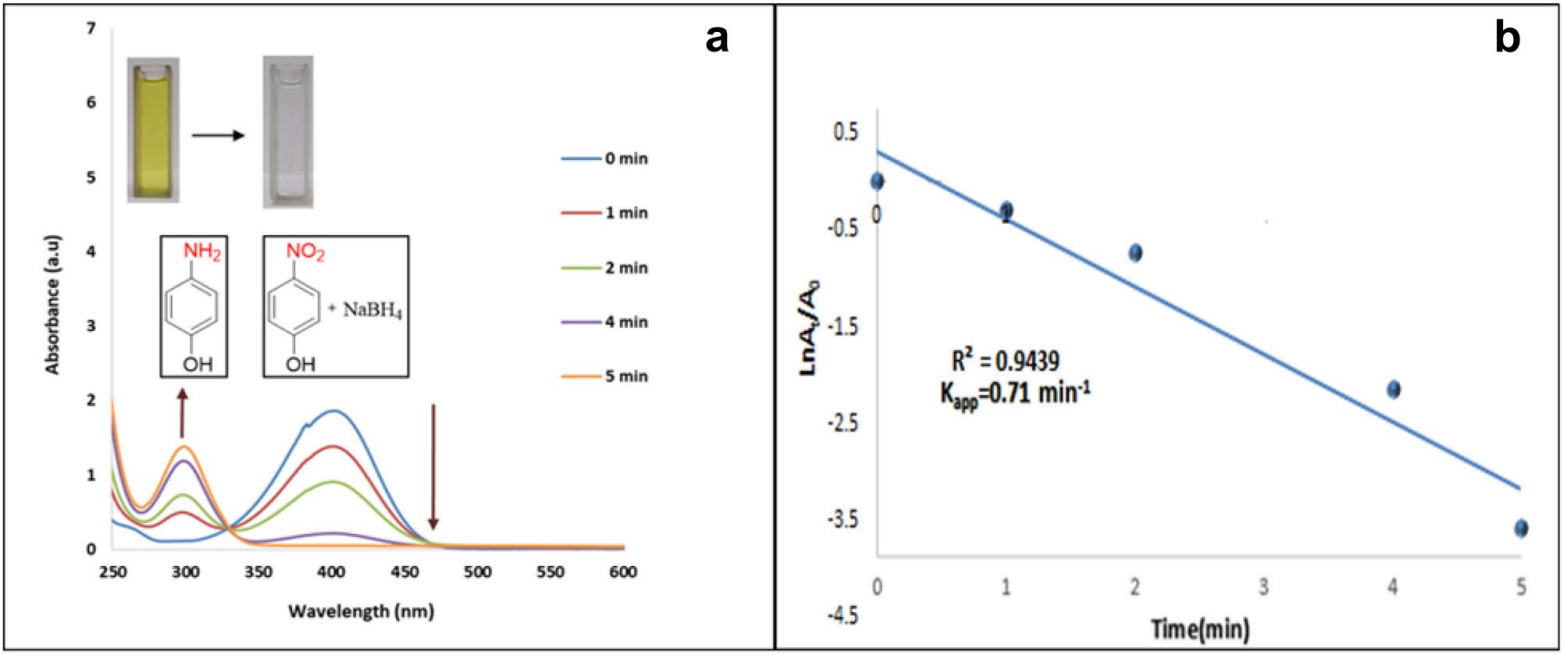
(**a**) UV–Visible spectra of the reduction of *p*-AP (0.5 mmol) over n-HA/Si-PA-SC@Ag (30 mg) and NaBH_4_ (7.5 mmol) in H_2_O (2.5 mL) at r.t. in different times. (**b**) Plot of ln (A_t_/A_0_) *vs.* time for the reduction of 4-NP dye.

For further study, the catalytic properties of the n-HA/Si-PA-SC@Ag was expanded and optimized in the reduction reaction of NAs, MB and CR with NaBH_4_. One of the most important of *p*-NP reduction is detection of the reaction product that the progress can be followed by detecting the changes in UV–Vis absorption at 400 nm and 300 nm. Based on the result of the model reaction, after 5 min the color of reaction changed to colorless from yellow (Fig. 4a). Immediately, catalyst was separated and a solution with certain molarity was prepared and its UV–Vis absorption was investigated. As presented in Fig. S7, the absorption peak at 400 nm was removed and a sharp peak at 300 nm demonstrating the presence of *p*-AP was appeared. Then, to select the best conditions for reduction of *p*-NP, the amount of H_2_O as a media, catalyst and NaBH_4_ were optimized and all results are tabulated (Table S3 and Fig. S8, S9, S10). Next, diverse amount of NaBH_4_ using n-HA/Si-PA-SC@Ag (20 mg) in the reduction of *p*-NP (0.5 mmol) in water (5 mL) at r.t. were studied (Table S3, entries 1–5 and Fig. S8). According to Fig. S8, the peak at 400 nm significantly reduced from 2.5 mmol (50 min) to 10 mmol (5 min) of NaBH_4_ and a new peak corresponds to *p*-AP was observed. This result indicates the reduction of *p*-NP produced absolutely *p*-AP, without any by-products with 100% yields while when 2.5 mmol of NaBH_4_ was used, only the *p*-AP was obtained with 40% yield. Based on experimental results from optimization of catalyst amount, almost similar times for *p*-NP reduction reaction were observed for 30 and 40 mg of n-HA/Si-PA-SC@Ag (Table S3, entries 6–9 and Fig. S9). Time-dependent changes in the absorption peak of *p*-NP at 400 nm occurred over n-HA/Si-PA-SC@Ag. The results of the reduction procedure in the absence of n-HA/Si-PA-SC@Ag and NaBH_4_ are presented in Table S3 and Fig. S10. As expected, color changes and desired products were not obtained without the use of n-HA/Si-PA-SC@Ag and NaBH_4_ over 2 h.

We also established the catalytic activity of catalyst for the reduction of other nitro-substates (Table 1). As tabulated and exhibited in Fig. S11 and based on mechanism in Fig. S12, it was found that our n-HA/Si-PA-SC@Ag catalyst promoted high reactivities for several nitroarenes bearing electron-donating and withdrawing groups and nitrobenzene. Notably, when *p*-nitrophenyl palmitate was used as a substrate, it not only did not lead to reduction, but also caused the breakdown of the bond between oxygen and carbonyl group and the production of *p*-AP (Table 1, entry 6). It should be mentioned that the structure of some products were confirmed by GC analysis. (see Figs. S18, S19,S20, S21 and S22in SI.).

**Table 1:** Synthesis of NAs catalyzed by n-HA/Si-PA-SC@Ag under optimized conditions ^a^.


Entry	Nitroarene	Aminoarene	Time (min:sec)	Conversion ^b^ (%)	Selectivity (%)
1			5:00	100	100
2			9:00	100	100
3			27:00	100	100
4			34:00	100	100
5^b^			53:00	100	100
6			2:40^´́^	100	100
7^b^			3:00	100	100
8^b^			6:00	100	100
9^b^			30:00	100	100
10^b^			5:00	100	100
11^b^			11:00	100	100

^a^ Reaction condition: *p*-NP (0.5 mmol), n-HA/Si-PA-SC@Ag (30 mg), NaBH_4_ (7.5 mmol) in H_2_O (2.5 mL) at r.t

^b^ H_2_O:EtOH/1.5:1 mL.

The reductive conversion of *p*-NP to *p*-AP is a six-electron transfer reaction in the presence of NaBH_4_ as a reducing agent, but will not proceed well in the absence of a catalyst. According to the reported mechanisms^59^, NaBH_4_ first produces nitro-phenolate ions, then BH_4_ (borohydride) and C_6_H_4_NO_3_^−^ (*p*-nitro-phenolate) ions are absorbed on the catalyst surface for electron transfer, and nitrophenolate ions absorb at 402 nm is significantly reduced and the reaction mixture is colorless. The reaction mechanism of the conversion of *p*-NP to *p*-AP in the presence of n-HA/Si-PA-SC@Ag is depicted in Fig. S12.

**Figure 5:**
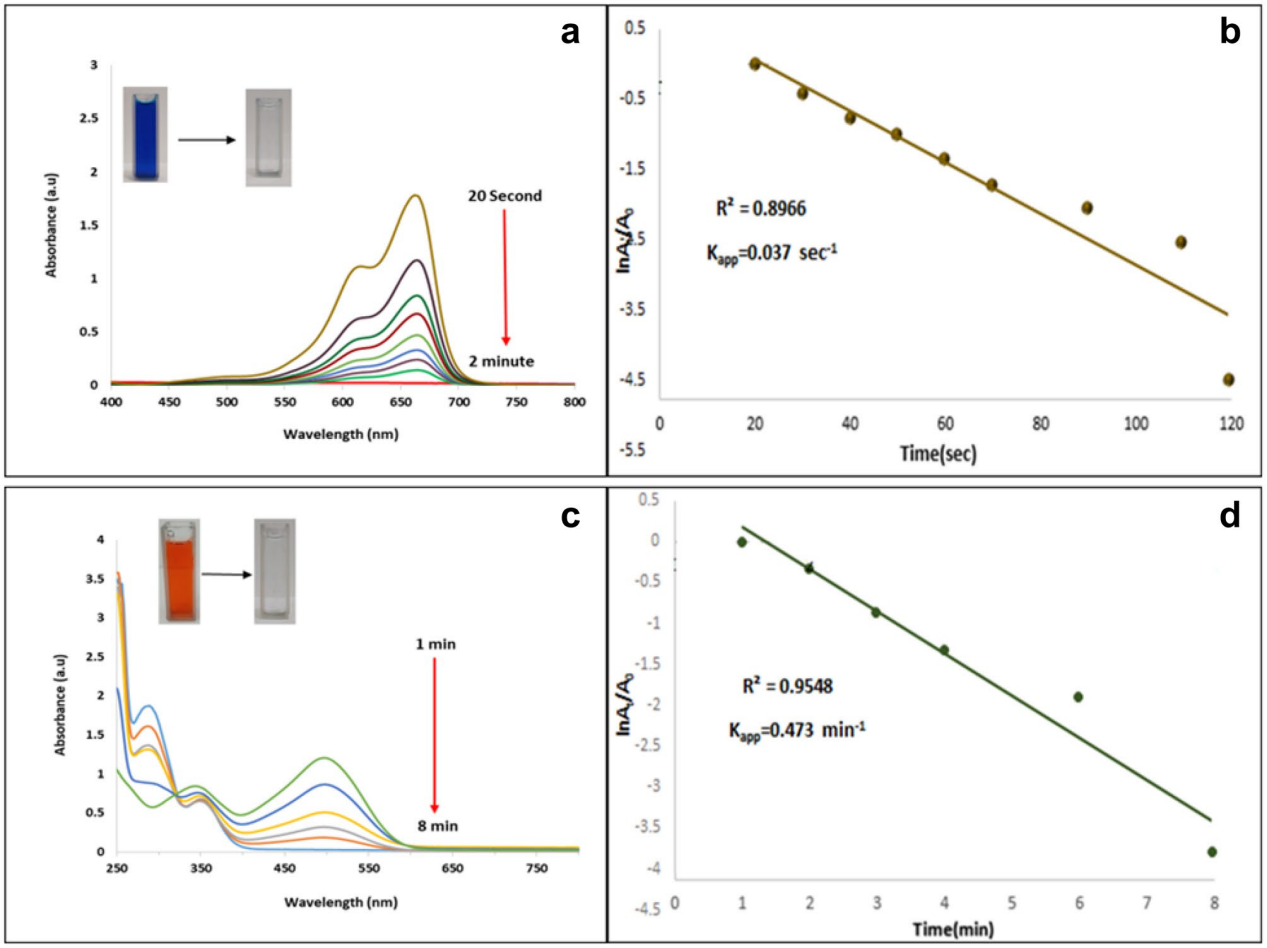
Reduction reaction of MB (**a**) and CR (**c**) by NaBH_4_ (10 mmol) and n-HA/Si-PA-SC@Ag (300 mg) in H_2_O at room temperature and plot of ln (A_t_/A_0_) against time for the reduction of MB dye (**b**) and CR dye (**d**).

Another possible application of synthesized n-HA/Si-PA-SC@Ag catalytic activity was the reduction of MB to LMB and CR to sodium-4-amino-1-naphtalene solfunate by NaBH_4_. For this porpuse, the reaction of MB or CR (0.5 mmol) over NaBH_4_ (10 mmol) and 300 mg of n-HA/Si-PA-SC@Ag in water at r.t. was started and the reaction progress followed by UV–Vis spectrophotometry in 400 and 800 nm. At the start of the reaction, the MB solution showed two peaks 664 and 614 nm^60^. After a few minutes of the reaction, these two peaks gradually decreased until after 2 min they completely disappeared and the color of reaction changed from blue to colorless, while this mixture did not change after two hours in the absence of n-HA/Si-PA-SC@Ag catalyst (Table 2, entry 1). The UV–Vis spectrum of the MB reduction by NaBH_4_ over n-HA/Si-PA-SC@Ag catalyst is shown in Fig. 5a. In agreement with proposed mechanism, the reduction process was establish to be enhanced over Ag nanoparticles and also exhibited a fast decrease in the absorption intensity of MB solution. In fact, Ag nanoparticles help in the electron relay from BH^−^_4_BH_4_^−^ as a nucleophilic core to MB as an electrophilic core (Fig. 5a)^61^.

**Table 2:** Reduction of MB and CR catalyzed by n-HA/Si-PA-SC@Ag.


Entry	Substrate	Catalyst (mg)	NaBH_4_ (mmol)	H_2_O (mL)	Conversation^b^ (%)	Time (min)
1	MB	300	10	5	100	2
2	CR	300	10	5	100	8

On the other side, the reduction reaction of CR (0.5 mmol) in the presence NaBH_4_ (10 mmol) and n-HA/Si-PA-SC@Ag (300 mg) in water at r.t. was investigated and the progress was checked by UV–Vis spectrophotometry in 250 and 800 nm. When the reaction was started, the CR solution showed two peaks 498 nm (*π* → π^*^) and 350 nm (*n* → π^*^), transition associated with the azo-group^62^. After a few minutes of the reaction, these two peaks gradually decreased until after 8 min they completely disappeared and the color reaction changed to colorless from red, while this mixture did not change after two hours in the absence of n-HA/Si-PA-SC@Ag catalyst (Table 2, entry 2). The UV–Vis spectrum of the CR reduction over n-HA/Si-PA-SC@Ag catalyst and NaBH_4_ is shown in Fig. 5c. In accordance with^63^, CR shows an absorption peaks that metal act as an electron relay, and electron transfer take place via Ag nanoparticles from BH^−^_4_BH_4_^−^ as a nucleophilic molecule to CR as an electrophilic molecule. Moreover, CR mixture and BH^−^_4_BH_4_^−^ ions in the presence of Ag nanoparticles was rapidly decolored representing the significant catalytic influence of Ag nanoparticles in the degradation of CR. The UV–Vis spectra of MB and CR exhibited a impressive decrease in peak strength because of the reduction by NaBH_4_ over n-HA/Si-PA-SC@Ag catalyst.

Additionally, it can be inferred that the reduction reaction follows pseudo-first-order kinetics because NaBH_4_ is usually consumed in excess compared to the concentration of nitrophenols and nanocatalysts. A few fundamental equations that give us information about the progress of a reaction are described below:

1 $$r=\frac{{\partial C}_{t}}{\partial t}=\mathrm{ln}\left(\frac{{C}_{t}}{{C}_{0}}\right)=-{K}_{app} t$$

2 $$r=\mathrm{ln}\left(\frac{{A}_{t}}{{A}_{0}}\right)=-{K}_{app}t$$

3 $$r=\mathrm{ln}\left(\frac{{A}_{t}}{{A}_{0}}\right)=\mathrm{ln}\left(\frac{{C}_{t}}{{C}_{0}}\right)=-{K}_{app}t$$

   The r, C_t_, C_o,_ A_t_ and A_o_ parameters represent the rate of reduction, concentration of nitro compound at any time t, initial concentration at zero time, absorbance at any time t, and the initial intensity of absorbance at time zero, respectively. Based on Eq. (3) it can be mention that absorption ratio of nitro phenols is equals to that of concentration ratio in reduction medium from any time t $=$ t to initial time t $=$ 0. Also, the apparent rate law can be easily calculated using by Eq. (2), that kaap is the apparent rate constant for first-order kinetics^64^.

The linear plots of the reduction of *p*-NP, MB and CR are demonestrated in Figs. 4b, 5b and 5d. As presented in Fig. 4b, a linear correlation was found between ln (*A*_*t*_/*A*_0_) and time and the calculated rate constant (*k*) was about 0.71 min^-1^^65^.The linear plot of ln (*A*_*t*_/*A*_0_) *versus* time shows that the reduction reaction followed the pseudo-first-order kinetics, and the calculated rate constant (*k*) was 0.473 min^−1^ (Fig. 5b). As shown in Fig. 5d, a linear correlation between ln (A_t_/A_0_) and time and the calculated rate constant (k) from the slope was 0.037 s^−1^. In the absorption process, the effect of contact time in different concentrations of *p-*NP, MB and CR dyes (0.031, 0.051, 0.073, and 0.095 mg/L) on the catalyst (0.28 mg/L) was investigated. The test solution for *p*-NP was performed at different time intervals (1, 2, 4, 5, 10, 20, 30, 40 and 60 min). The absorption range of *p*-NP was found from 80 to 100% in the studied concentrations of 0.031, 0.051 and 0.073 mg/L (Fig. S13c) and the absorption range of MB and CR dyes were found from 80 to 100% in the studied concentrations of 0.031 and 0.051 mg/L (Fig. S13a and b) that the absorption rate was faster in MB. Based on the results obtained, the color removal rate decreased with increasing color concentration from 0.031 to 0.095 mg/L, which was the same for all three samples. The reason for that was the less availability of binding sites in blue dye solutions.

In order to investigate the activity of the n-HA/Si-PA-SC@Ag catalyst as much as possible, the reduction reactions of *p*-NP, MB and CR were investigated in the absence of the catalyst and the results were compared with each other. As seen in Fig. S14, S15 and S16 and according to the mechanism, nitro compounds need H^-^ for reduction processes, which is provided from NaBH_4_ salt, but the presence of Ag is very effective and acts as a catalyst to advance the reaction and complete it. This reaction is completed in the presence of hydrogen source and catalyst and is incomplete in the absence of any of these. As shown in Fig. S14, when the catalyst alone is present in the reaction mixture, we have more than 50% of the amine product, while in the absence of the catalyst, there is no reduction in the reaction mixture. Furthermore, the dependence of the reduction reaction on the hydrogen source is clearly visible in the reduction of MB and CR dyes, but the presence of the catalyst is also effective (Fig. S15 and S16).

For investigation of the further proficiencies, the performance of the n-HA/Si-PA-SC@Ag in the reduction of *p*-NP, MB and CR were compared with other recent reports (Table S4, entries 1–14). As tabulated, it was determined that AAs were achieved in better reaction condition and shorter reaction time by using n-HA/Si-PA-SC@Ag (Table S4, entry 6). Actually, the biggest advantage of the above catalyst is achieving the highest yields in the shortest reaction times along with easy separation. Among other advantages of this manner we cat mention, green nature of n-HA/Si-PA-SC@Ag, eco-friendly process, easy workup procedure and high product's yield that are formed in mild conditions.

### Recyclability study

Based on the importance of recycling modern catalysts in their applied use, the ability of n-HA/Si-PA-SC@Ag in the synthesis of AAs through the reduction reaction was studied. For this end, upon completion of the reaction, n-HA/Si-PA-SC@Ag was filtrated, washed with H_2_O/ethanol and used for the next run under the same condition. As can be seen that in Fig. S17C, this series was repeated up to four repeated times without any decrease in activity that verified by Ag leaching results (0.0010 mmol.g^−1^). Regarding to the results obtained from Fig. S17C, the UV–Vis spectra of the products of each stage of the reduction reaction over the recycled n-HA/Si-PA-SC@Ag investigated. Surprisingly, the results confirm that *p*-AP was obtained with 100% yield without any side-products (Fig. S17D). As delineated in Fig. S17B, the stability of the structure of recycled n-HA/Si-PA-SC@Ag was studied using recording FTIR analysis after one and last runs. Obviously, all spectra are similar and no momentous changes were detected upon recycling, which this observation was matched with the results obtained from SEM analysis (Fig. S17A).

## Experimental

### Materials and instruments

For this project, some chemical and reagents purchased from Sigma-Aldrich, including, diammonium hydrogen phosphate, calcium nitrate tetrahydrate, NaBH_4_, NH_3._H_2_O, TEOS, EtOH, toluene, Et_3_N, (3-chloropropyl) trimethoxysilane, phthalimide, semicarbazide, nitro compounds, MB, CR, MeOH, deionized water and AgNO_3_ and following the reaction progress were done by UV–Vis, GC and TLC on aluminum-backed plates of silica gel 60 F254.

The catalyst characterization was accomplished using, XRD, BET, FTIR, TGA, SEM/EDX, TEM and ICP-AES. The instrument for FTIR and UV–Vis spectra were PERKIN-ELMER-spectrum 65. All patterns of XRD were achieved on a Rigaku Ultima (Japan), operating at 20–60 kV and 2–60 Ma at r.t. SEM/EDX and TEM images were found via a Tescan instrument using Au-coated samples (20 kV) and Philips EM 208 s instrument, respectively. Thermal gravimetric analyses were recorded by a TA instrument; model Q600 from room temperature to 1350 °C (Rate: 20 °C min^−1^), under N_2_ atmosphere. For investigation of textural properties of catalysts (BET), BELSORP Mini II instrument were carried out and both samples degassed at 423 K for 1.5 h. Moreover, experimental research and field studies on plants were in compliance with institutional guidelines.

### Synthesis of n-HA/Si-PA-SC@Ag

#### Synthesis of n-HA

First, two aqueous solution of HPO_4_[NH_4_]_2_ (0.65 M, 50 mL) and Ca(NO_3_)_2_.4H_2_O (1.8 M, 50 mL) were prepared, mixed together and refluxed at 100 °C under Ar (0.5 h). Then, NH_3_.H_2_O (about 10 mL) was slowly injected to mixture until the pH reached to 11 and then refluxed for more 24 h. Afterward, the mixture filtered, washed with H_2_O and dried in oven at 55 °C overnight.

#### Synthesis of n-HA/Si

In this step, 6 g of n-HA was dispersed in EtOH/H_2_O (50/17 mL) for half time. Then, NH_3_.H_2_O was added to the above-mixture up to the pH reach to 11. After half an hour, TEOS (12 g in EtOH 30 mL) was added and stirred for 12 h. Finally, n-HA/SiO_2_ was achieved after filtration, washing with H_2_O and EtOH and drying in oven at 50 °C for 6 h.

#### Synthesis of n-HA/Si-Cl

To synthesize the n-HA/Si-Cl, n-HA/Si (1 g) was dispersed in toluene (35 mL) for 30 min. After that, the mixture was heated at refluxed under Ar and (3-chloroprppyl) trimethoxysilane (7 mL) and Et_3_N (1.3 mL) were added into the mixture and its pH was kept at 8.5. After overnight, the resulting mixture filtered, washed with toluene and dried at 45 °C for 6 h.

#### Synthesis of n-HA/Si-PA

Regarding to synthesis n-HA/Si-PA, n-HA/Si-Cl (3 g) dispersed in toluene (60 mL) under U.I. for 30 min and a solution of phthalimide (2.22 g in 60 mL toluene:CH_3_CN/ 2:1) and Et_3_N (1 mL) were added to the mixture and its pH was ~ 8.2. refluxed under Ar, overnight. Finally, the obtained result was filtrated, washed with toluene and dried in 60 °C for 12 h.

#### Synthesis of n-HA/Si-PA-SC

N-HA/Si-PA (5 g) was dispersed in 70 mL toluene under U.I. for 30 min. Then, a solution of semicarbazide (5.1 g in 90 mL methanol and toluene/ 2:1) was prepared and added into the above mixture with Et_3_N (~ 2 mL) and refluxed and Ar for 24 h (pH ~ 9). Lastly, the mixture was filtered, washed with toluene/methanol and dried at 40 °C in an oven.

#### Plant material and extract preparation

The plant was collected in April 2011, in Siahkal, Gilan, Iran. The voucher specimen has been identified by Dolatyari, Ramezani and Ajani and deposited at the Flora of Iran Herbarium of Iranian Biological Resources Center (Collection number IBRC P1006947).

The flowers (10 g) collected from *Bellis perennis L., Asteraceae* and then crushed in porcelain mortar and turned into a uniform powder. To the obtained powder, water (100 mL) was added and heated for 2 h at 100 °C. When the color solution becomes dark and its volume is half, the extract was filtrated and it was used for the reduction of metal salts (pH ~ 10.2).

#### Synthesis of n-HA/Si-PA-SC@Ag

**Figure 6:**
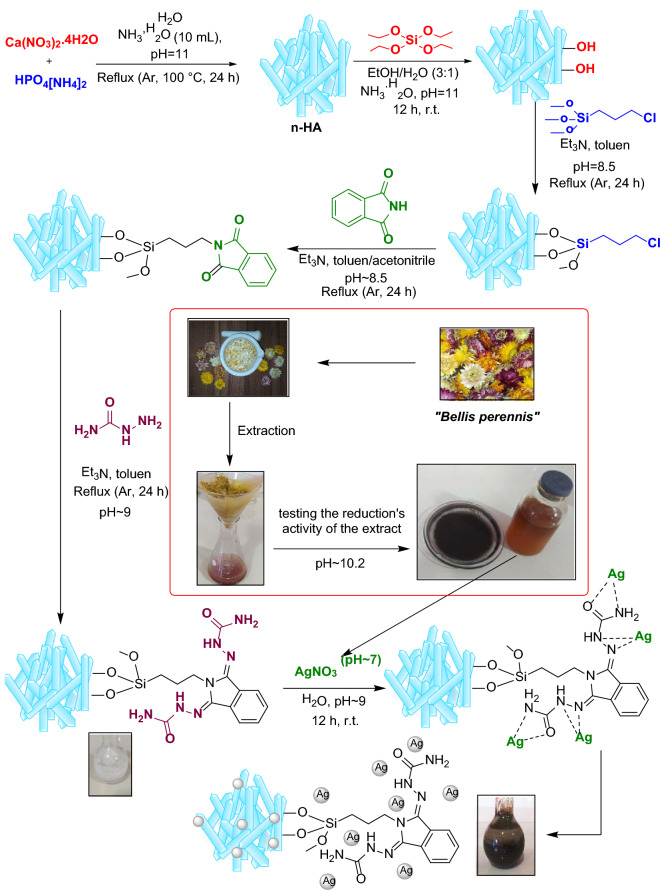
The synthetic process of n-HA/Si-PA-SC@Ag.

In the last step, AgNO_3_ salt was incorporated onto the n-HA/Si-PA-SC via bio-assist approach. First, n-HA/Si-PA-SC (3 g) was dispersed in H_2_O (35 mL) for 30 min and stirred under Ar. After that, AgNO_3_ (0/09 g in 10 mL of H_2_O, pH ~ 7) solution was prepared and added into the above mixture and stirred for more 30 min. Then, the fresh extract (10 mL) was dropwise added and the mixture stirred for 4 h. Obviously, the color of the mixture was changed from white to black, which this observation caused by the reduction of Ag(II) salt to Ag(0)-NPs. Notably, after adding fresh extract to the mixture including AgNO_3_, the pH was increased to ~ 9. Eventually the mixture was filtered, washed with H_2_O/EtOH and dried in oven at 60 °C for 15 h and then the gray powder was achieved (Fig. 6).

### Catalytic activity

#### General method for the reduction of NAs

For investigation of the catalytic activity, in a typical manner in a solution of NAs (0.5 mmol, in 1 mL H_2_O), a solution of NaBH_4_ (7.5 mmol, in 1.5 mL H_2_O) and n-HA/Si-PA-SC@Ag (30 mg) as a catalyst were added and stirred at r.t. (pH ~ 9). The reaction process was checked by UV–Vis and TLC. Upon completion of this reduction (changing the mixture's color from yellow to colorless), n-HA/Si-PA-SC@Ag was filtrated, washed and dried for using next run. Finally, a solution (0.01 M, 5 mL) from concentrated was prepared and the purity percentage and yield of the product were checked by UV–Vis and GC analyses.

#### General method for the reduction of MB and CR

Two solution of MB and CR (0.5 mmol in 3 mL H_2_O) were prepared, separately. Then 300 mg n-HA/Si-PA-SC@Ag and a solution of NaBH_4_ (10 mmol in 2 mL H_2_O) were added to them and both were stirred until they became colorless (pH ~ 10.5–11). Upon completion of the reduction, the reaction process was followed by UV–Vis. After completion of the reduction (changing the color of the reaction mixture blue and orange to colorless), n-HA/Si-PA-SC@Ag was filtrated, washed and dried for using next run. Finally, a solution (0.2 M, 25 mL) from concentrated was prepared and the purity percentage and yield of the product were checked by UV–Vis analysis.

## Conclusion

As a whole, a natural-based and heterogeneous catalyst has been developed by the preparing and functionalizing of n-HA with phthalimide, semicarbazide and Ag nanoparticles that were incorporated via bio-assisted approach using *BP*E. The structure of synthesized catalyst with stability and suitability of the synthesis method were confirmed. The performance of n-HA/Si-PA-SC@Ag for the reduction of NAs, MB and CR were investigated. n-HA/Si-PA-SC@Ag catalyst is not only effective in the reduction of NAs but also shows outstanding activity in the reduction of organic dyes. Besides to its high reactivity, the above catalyst was easy separated and recycled up to four more times. Noteworthy, the use of n-HA as a solid substrate has a significant and positive effect on immobilizing Ag and suppressing its leaching. Likewise, the DFT and QAITM computational results were consistent with that of experimental observations from the structural and electronic point of view.

## Supplementary Information

Supplementary Information.

## Data Availability

All data generated or analysed during this study are included in this published article (and its Supplementary Information files).
