# Supplementary material for: Direction of theoretical and experimental investigation into the mechanism of n-HA/Si-PA-SC@Ag as a bio-based heterogeneous catalyst in the reduction reactions
Source: Sci Rep. 2022 Dec 19;12:21964. doi: 10.1038/s41598-022-26200-3 (PMC9763413; doi:10.1038/s41598-022-26200-3)
Supplement: Supplementary file 1 — Supplementary Information. [file 41598_2022_26200_MOESM1_ESM.pdf]

Supporting information

Results and discussion

Catalyst characterization

At first, the general structures of stepwise of catalyst synthesis (A-F) were identified by FTIR analysis and demonstrated in Fig. S1. As presented in Fig. S1A and accordance with the previous literature, [1] several bands at 565, 576 and 1035 cm^{-1} that can be recognized to PO_4^{3-} and the band at 3566 cm^{-1} (-OH group) confirms the n-HA structure. In Fig. S1B, the presence of SiO_2 in catalyst's structure established by observed two bands at 565 and 1092 cm^{-1} (Si-O-Si and Si-O stretching) [2]. In the Fig. S1D, the band at 1760 cm^{-1} is visible that can be assigned to –C=O group in the phthalimide (PA). Moreover, the conjugation of PA with semicarbazide (SC) in Fig. S1E, was established by detected some new bands at 1730 cm^{-1} (CONH$_2$ group), 1689, 1537 and 1388 cm^{-1} (C=N, C-N and C-C), 3330 and 3426 cm^{-1} (-NH$_2$). As shown in Fig. S1F, not only the corresponding bands of n-HA and amide linkage were appeared, but also a right shift to lower wavelength of amide bands and softening the intensity of amine peaks due to the interaction of the –NH$_2$ and oxygen in –C=O group with Ag-NPs were performed [3].

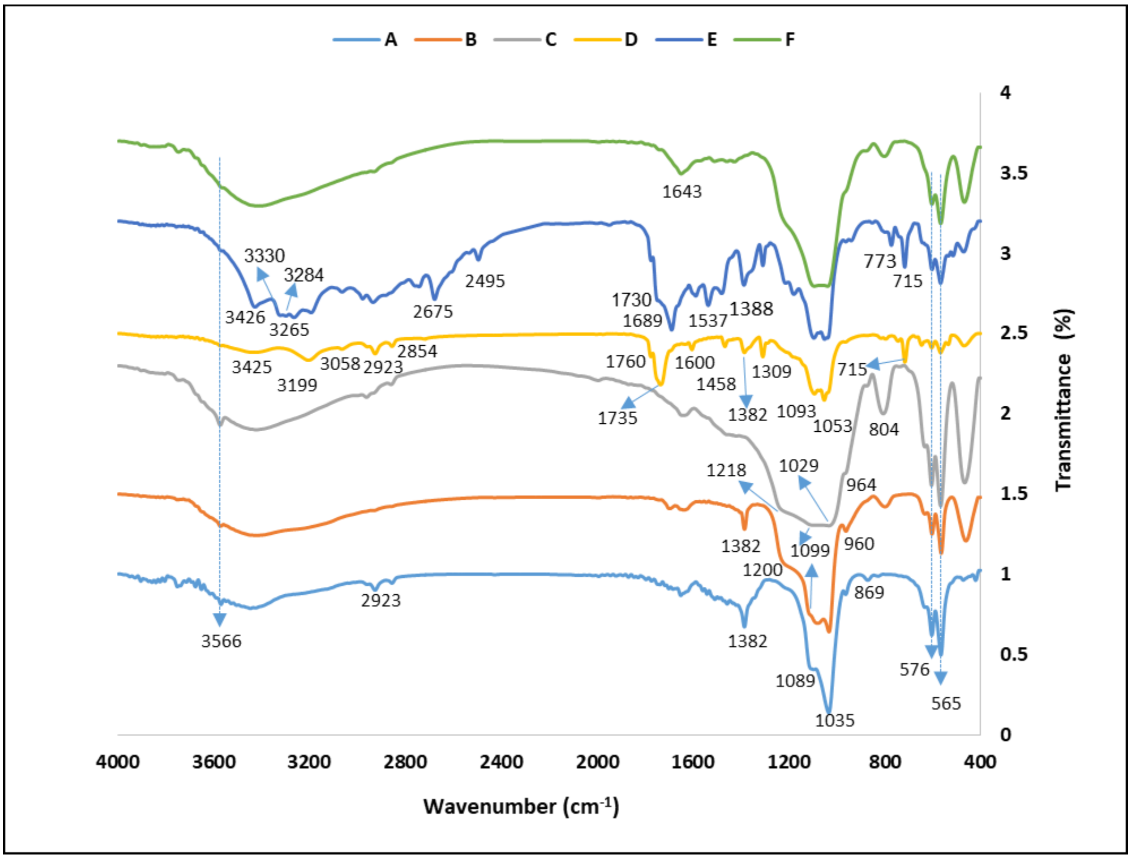

Figure S1. FTIR spectra of the n-HA (A), n-HA/Si (B), n-HA/Si-Cl (C), n-HA/Si-PA (D), n-HA/Si-PA-SC (E), n-HA/Si-PA-SC@Ag (F).

In agreement with XRD of n-HA (Fig. S2A), several characteristic bands (labeled as black dot) at 2θ = 26.38° (002), 28.12° (102), 29.08° (210), 31.93° (211), 33.16° (112), 34.05° (300), 39.85° (310), 47.23° (312), 48.08° (320), 49.43° (213), 50.02° (321) and 53.19° (004) in XRD pattern of n-HA/Si-PA-SC@Ag were detected. In accordance with previous literature [4], it can be decided that n-HA with crystalline structure has well preserved its structure after modification. Moreover, several other peaks at 2θ = 38.06° (111), 44.18° (200), 64.7° (220) and 77.57° (311) marked with asterisks can be evidently seen in Fig. S2B, which prove the presence of Ag nanoparticles. These peaks are completely consistent with the *fcc* (faced center cubic) crystalline structure of Ag nanoparticles (JCPDS card no. 04-0784) [5]. These notes correctly confirm that the n-HA surface is well covered by Ag nanoparticles and this surface modification has been successful.

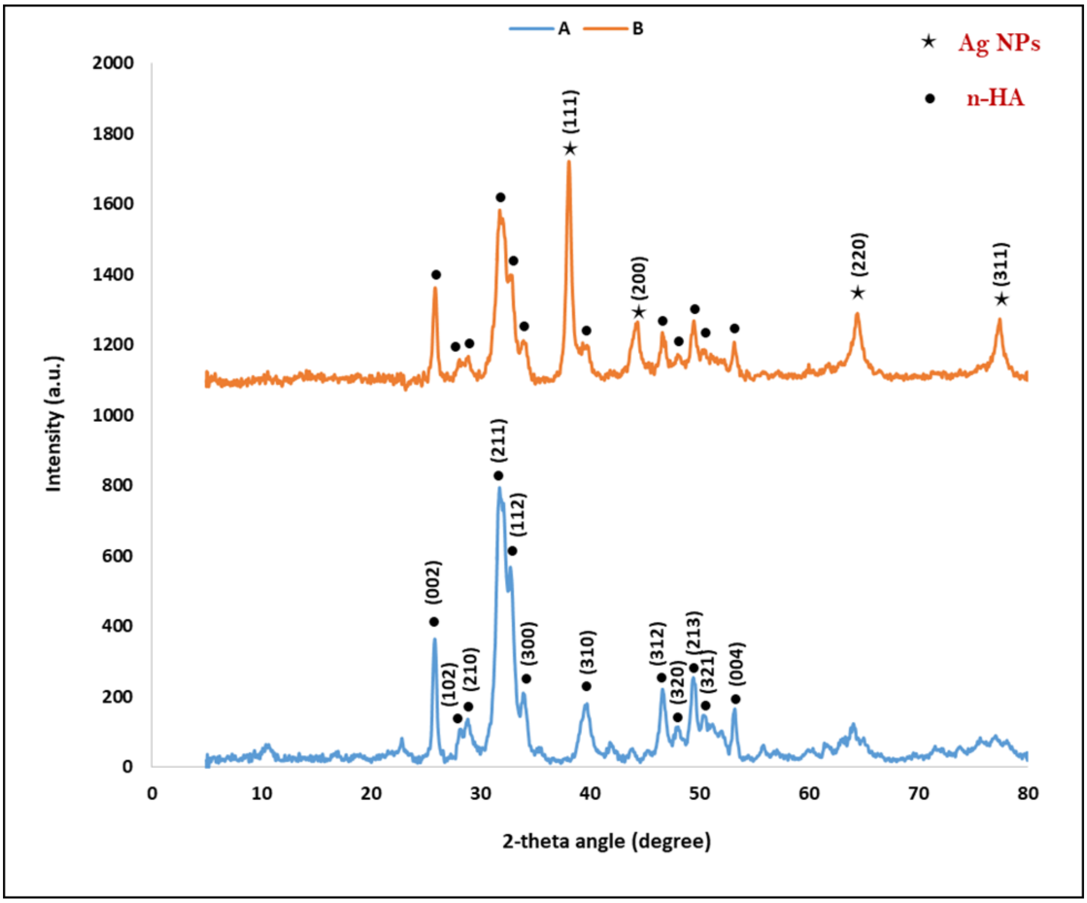

Figure S2. The XRD patterns of pure n-HA (A) and n-HA/Si-PA-SC@Ag (B).

The TG analyses of A) n-HA and B) n-HA/Si-PA-SC@Ag were recorded and their results described in Fig. S3. Based on the n-HA thermogram, two weight losses were exhibited over 45-700 °C that primary degradation occurred up to 200 ºC that is corresponds to the loss of H_2O and moisture. After that, the decomposition is very slow up to 700 °C with 3.4% weight loss that might be due to the progressive hydroxylation of n-HA. In TG analysis of n-HA/Si-

PA-SC@Ag three degradation stages at 45-700°C were exhibited. The first loss occurred up to 70 °C and then it remained constant up to 105 °C which may be because of water and moisture (3.9% w/w). The next loss happened up to 250 °C can be attributed to dehydroxylation of the n-HA (7.2% w/w). Finally, the last degradation was occurred up to 250 °C can be endorsed to the decomposition of inorganic materials in catalyst structure and organic motif (8.1% w/w). A total weight loss of 19.2% was perceived at 700 °C. Therefore, based on the results of the TG analysis, it can be admitted that the catalyst is stable thermally at the reaction temperature.

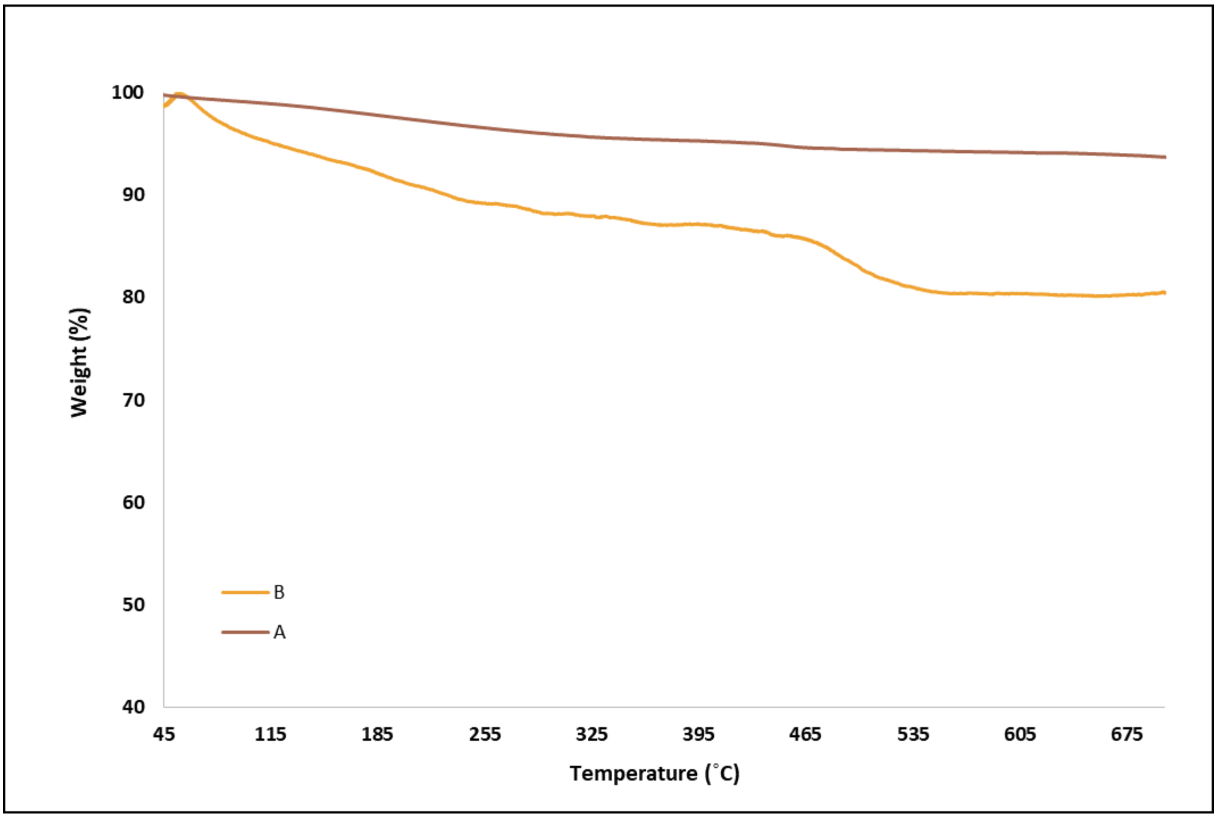

Figure S3. TG analyses of pure n-HA (A) and n-HA/Si-PA-SC@Ag (B).

The texture characteristics of n-HA/Si-PA-SC and n-HA/Si-PA-SC@Ag were determined by BET analysis. BET surface area of n-HA/Si-PA-SC and n-HA/Si-PA-SC@Ag are 11.07 and 94.54 m^2g^{-1}, respectively that are presented in Table S1. As show in Fig. S4 and according to IUPAC isotherm is IV type with mesoporous structure [6]. As tabulated, a considerable increase of all parameters occurred upon functionalization. This observation could be due to the production protocol that severely corroded the surface of n-HA/Si-PA-SC, yielding a porous material. In details, with reducing the Ag salt to Ag nanoparticle on the n-HA/Si-PA-SC by natural extract as reducing agent, the surface area increases.

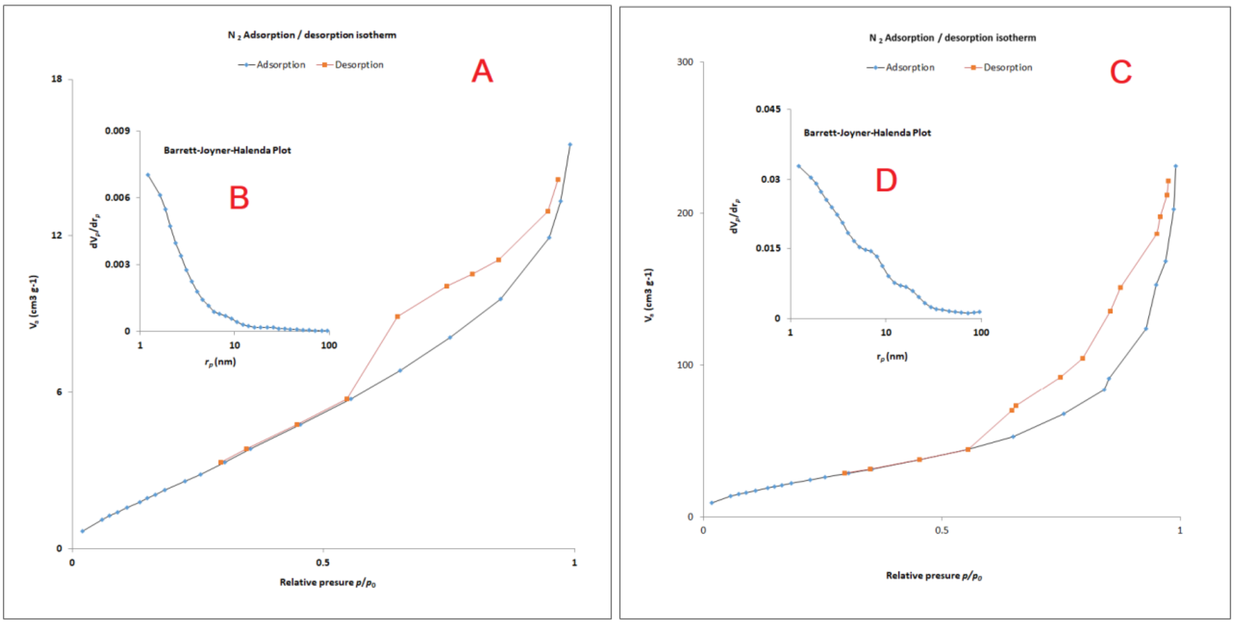

Figure S4. The N_2 adsorption-desorption isotherms (A, C) and BJH plots (B, D) of n-HA/Si-PA-SC, n-HA/Si-PA-SC@Ag.

Table S1. Textural properties of n-HA/Si-PA-SC and n-HA/Si-PA-SC@Ag.

**Catalyst**	**S_{BET} (m^2g^{-1})**	**Total pore volume (cm^3g^{-1})**	**Average pore diameter (nm)**
n-HA/Si-PA-SC	11.072	0.023716	8.568
n-HA/Si-PA-SC@Ag	94.542	0.3496	14.79

Based on the images obtained from SEM analysis (Fig. S5A-C), it can be visibly realized that the structure of n-HA is rod-like morphology, that this structure has been maintained in all stages of catalyst synthesis, and multi-step functionalization has been prosperous and no momentous change occurred in the body of n-HA. Moreover, the EDS analysis possesses C (9.27%), O (25.39%), Si (0.18%), P (14.97%) and Ca (50.18%) elements that confirms the effective formation of n-HA (Fig. S5A). The EDS result of n-HA/Si-PA-SC exhibited, C (10.91%), N (12.31%), O (29.73%), Si (13.53%), P (8.73%) and Ca (24.79%) elements that the presence of nitrogen atom confirms the successful functionalization (Fig. S5B). Based on Fig. S5C, the percentage of C (25.96), N (5.40), O (33.78), Si (10.19), P (6.31), Ca (13.97) and Ag (4.40) elements were obtained representing the structure of n-HA/Si-PA-SC@Ag. By comparing the results obtained from the n-HA/Si-PA-SC@Ag and n-HA/Si-PA-SC and also observing the decrease in the percentage of N, C, O, Si, P and Ca elements and the presence of Ag, it can be concluded that silver nanoparticles are well covered on the catalyst surface. On the other hand, Ag loading was achieved by ICP-OES analysis and calculated to be 0.013 $mmol.g^{-1}$.

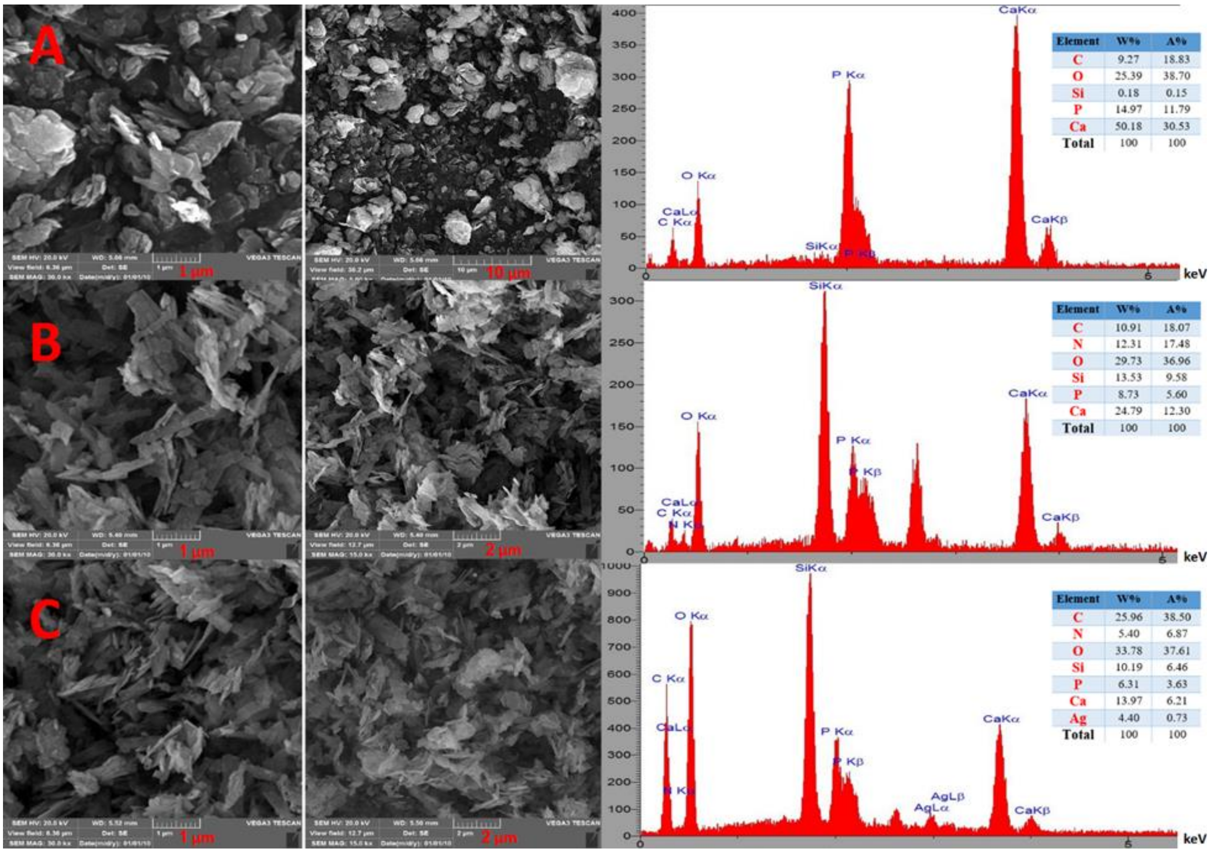

Figure S5. SEM-EDX images of n-HA (A), n-HA/Si-PA-SC (B) and n-HA/Si-PA-SC@Ag (C).

Computational section

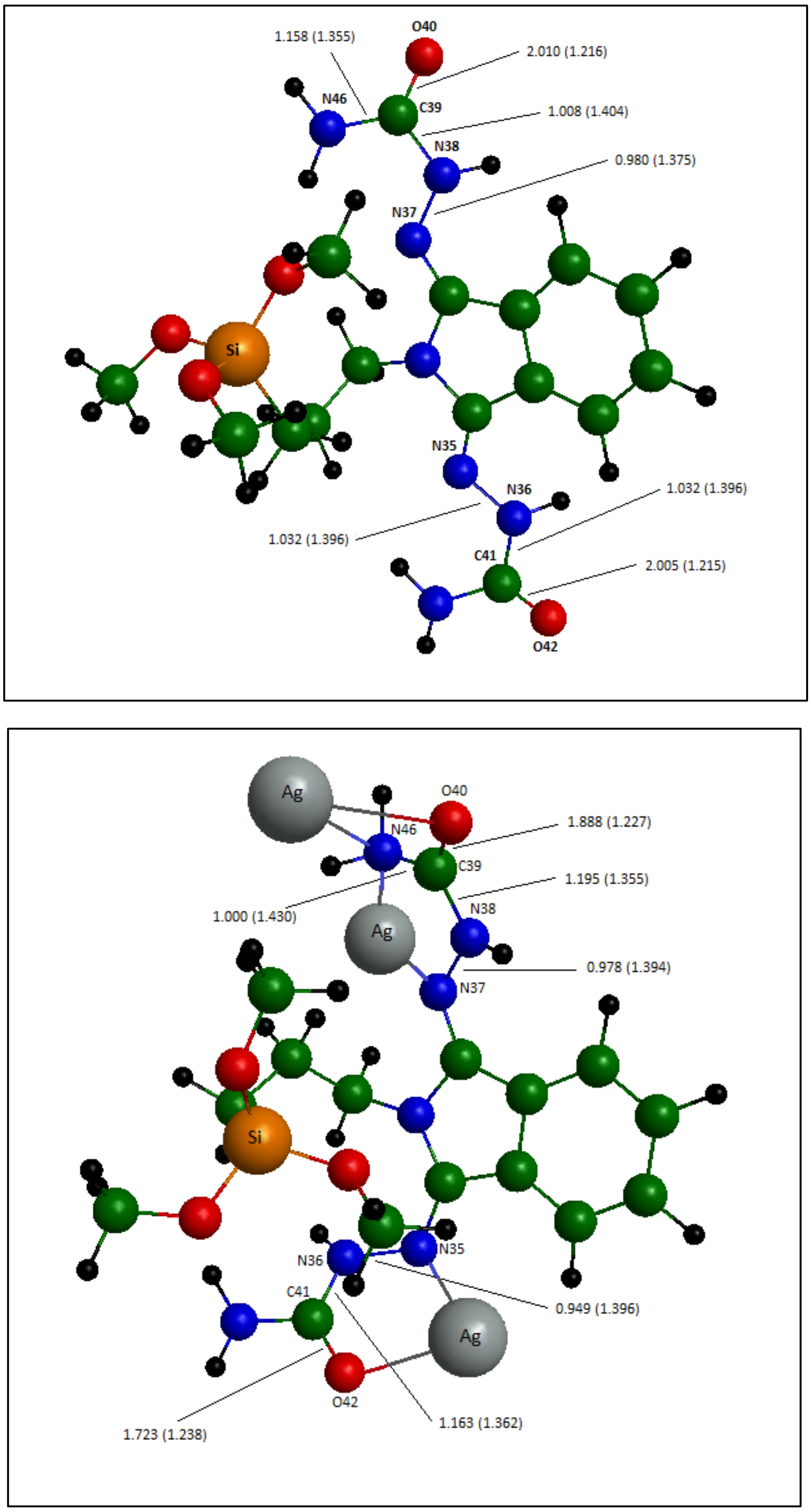

Figure S6. The optimized geometry of n-HA/Si-PA-SC ligand and n-HA/Si-PA-SC-Ag complex models. The calculated values of some selected key bond orders (bond lengths) at M06/6-311G** level of theory have been reported.

Table S2. QTAIM properties of some selected bond critical points (BCPs) in n-HA/Si-PA-SC ligand and n-HA/Si-PA-SC-Ag complex, obtained via wave function analysis at M06/6-311G** level of theory. Note that numbering of atoms is in accordance with Fig. 3.

	ρ_b	$\nabla^2\rho_b$	G_b	V_b	H_b	$\lvert V_b \rvert / G_b$
**n-HA/Si-PA-SC Ligand**						
**BCP(C39-N46)**	0.323	-0.954	0.186	-0.612	-0.426	3.290
**BCP(C39-O40)**	0.410	-0.426	0.581	-1.270	-0.689	2.185
**BCP(N38-C39)**	0.297	-0.854	0.146	-0.506	-0.360	3.465
**BCP(N37-N38)**	0.337	-0.565	0.165	-0.471	-0.306	2.854
**BCP(N35-N36)**	0.334	-0.552	0.162	-0.463	-0.301	2.858
**BCP(N36-C41)**	0.301	-0.866	0.153	-0.524	-0.371	3.424
**BCP(C41-O42)**	0.411	-0.419	0.585	-1.274	-0.689	2.177
**n-HA/Si-PA-SC-Ag Complex**						
**BCP(C39-N46)**	0.287	-0.789	0.117	-0.432	-0.315	3.692
**BCP(C39-O40)**	0.399	-0.375	0.568	-1.230	-0.662	2.165
**BCP(N38-C39)**	0.323	-0.919	0.208	-0.647	-0.439	3.110
**BCP(N37-N38)**	0.321	-0.491	0.157	-0.438	-0.281	2.789
**BCP(N35-N36)**	0.319	-0.487	0.154	-0.430	-0.276	2.792
**BCP(N36-C41)**	0.321	-0.945	0.186	-0.609	-0.423	3.274
**BCP(C41-O42)**	0.389	-0.456	0.521	-1.156	-0.635	2.218
**BCP(Ag55-O40)**	0.021	0.068	0.018	-0.019	-0.001	-1.055
**BCP(Ag55-H47)**	0.017	0.045	0.011	-0.012	-0.001	-1.090
**BCP(Ag55-H3)**	0.011	0.035	0.008	-0.007	0.001	-0.875
**BCP(Ag56-N46)**	0.029	0.090	0.025	-0.029	-0.004	-1.160
**BCP(Ag56-N37)**	0.029	0.100	0.028	-0.031	-0.003	-1.107
**BCP(Ag56-H25)**	0.020	0.058	0.016	-0.017	-0.001	-1.062
**BCP(Ag57-O42)**	0.047	0.232	0.059	-0.060	-0.001	-1.016
**BCP(Ag57-N35)**	0.055	0.239	0.065	-0.071	-0.006	-1.092
**BCP(Ag57-H51)**	0.025	0.080	0.022	-0.024	-0.002	-1.090
**BCP(Ag57-H14)**	0.015	0.052	0.012	-0.012	0.000	-1.000

Catalytic activity

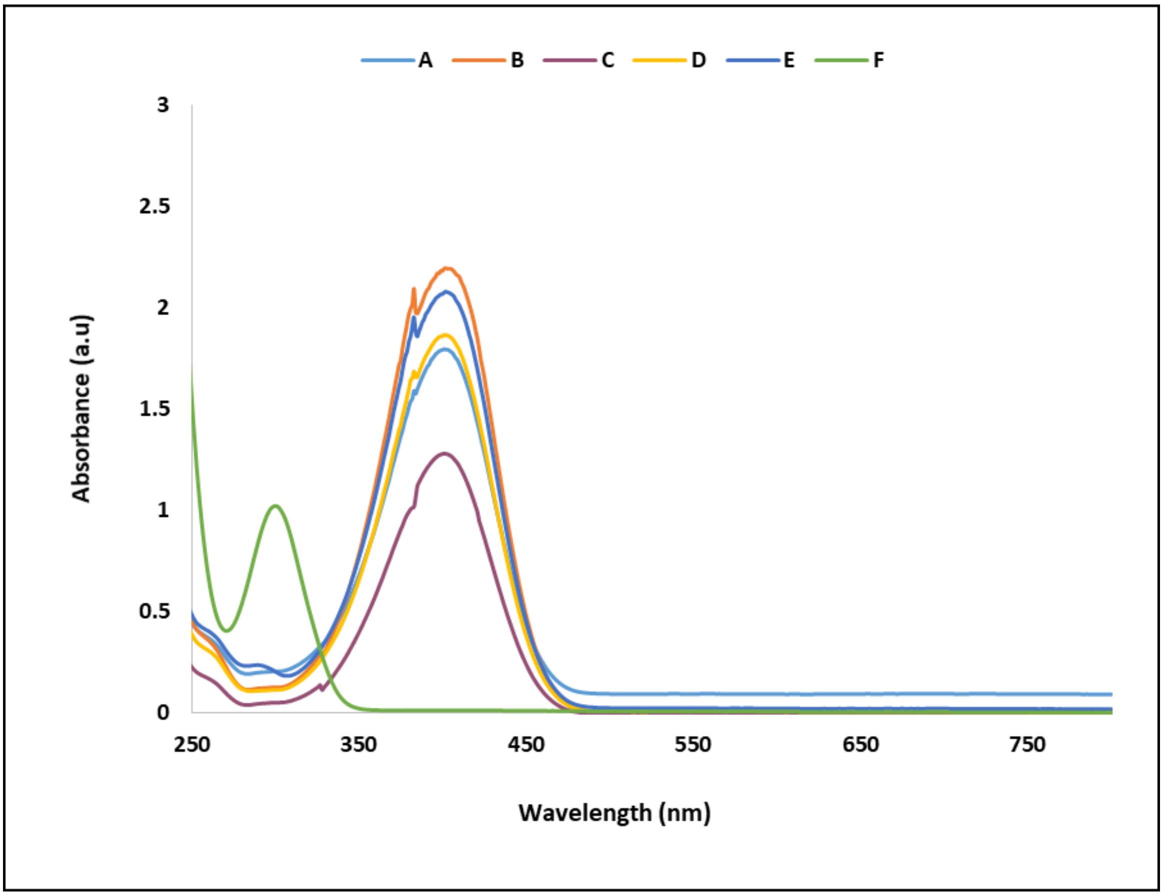

Figure S7. UV-Visible spectra of the reduction of *p*-NP over n-HA (A), n-HA@SiO_2 (B), n-HA@SiO_2-Cl (C), n-HA@SiO_2-PA (D), n-HA@SiO_2-PA-SC (E), Ag@n-HA@SiO_2-PA-SC (F) using $NaBH_4$ (7.5 mmol), in H_2O (2.5 mL) at r.t. during 5 minutes.

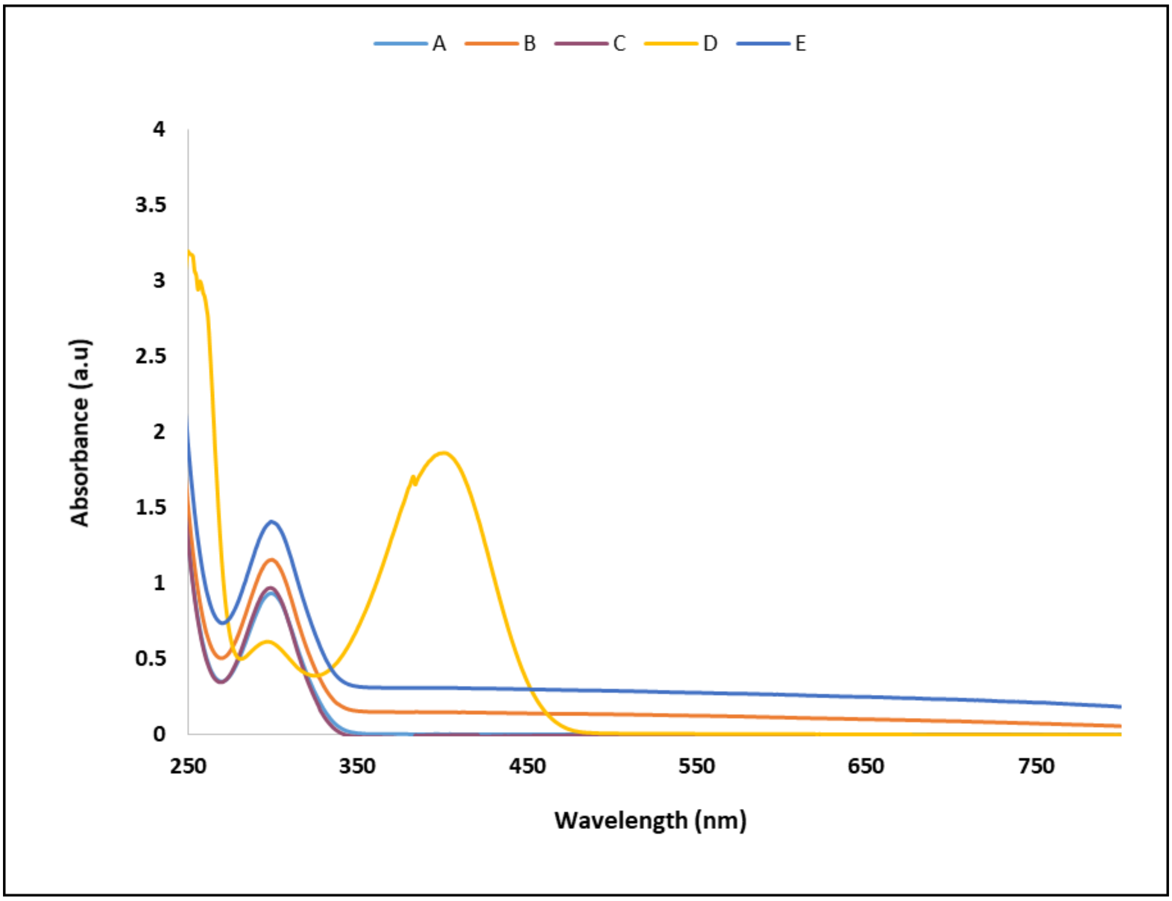

Figure S8. Optimization of amount of $NaBH_4$ in the presnce of n-HA/Si-PA-SC@Ag (20 mg), in H_2O (5 mL), at room temperature. A (10 mmol), B (7.5 mmol), C (5 mmol), D (2.5 mmol) and E (8.5 mmol).

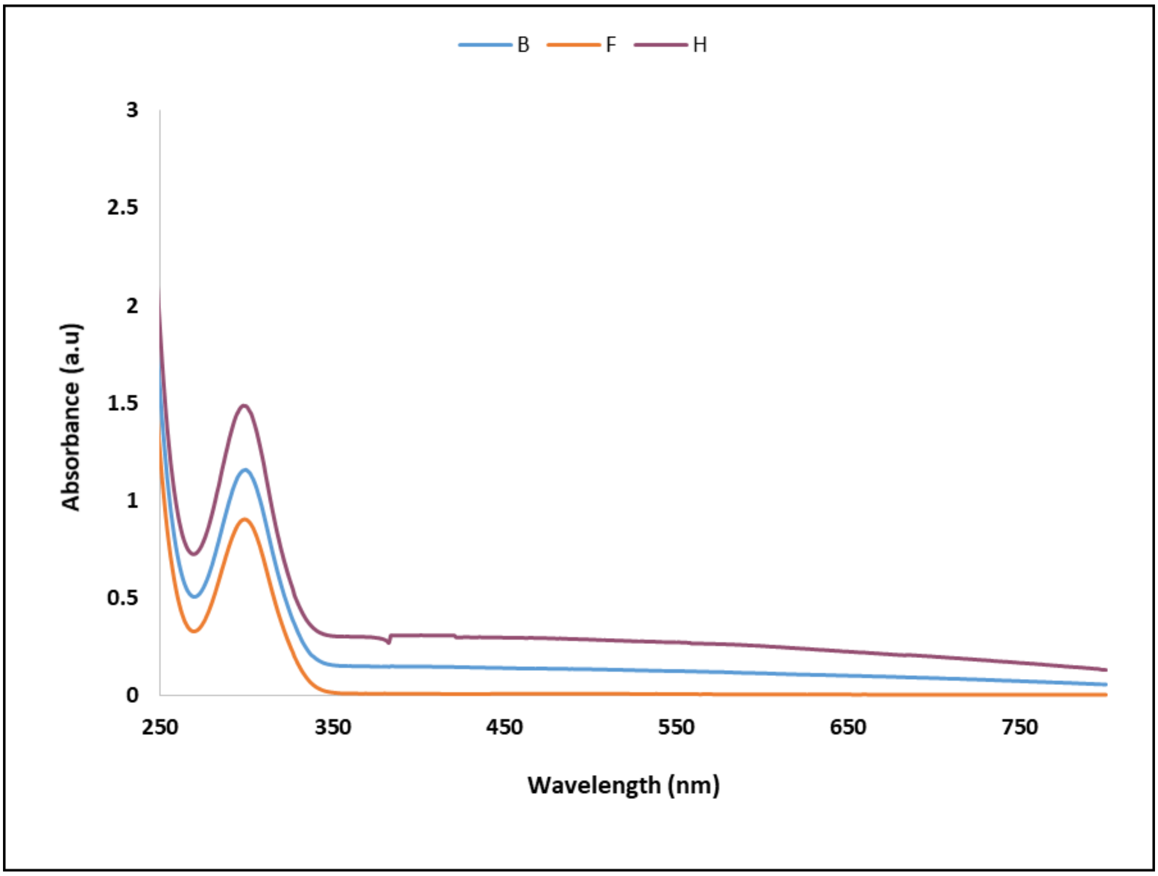

Figure S9. Optimization of amount of n-HA/Si-PA-SC@Ag in the presnce of $NaBH_4$ (7.5 mmol), in H_2O (5 mL), at room temperature. B (20 mg), F (30 mg) and H (40 mg).

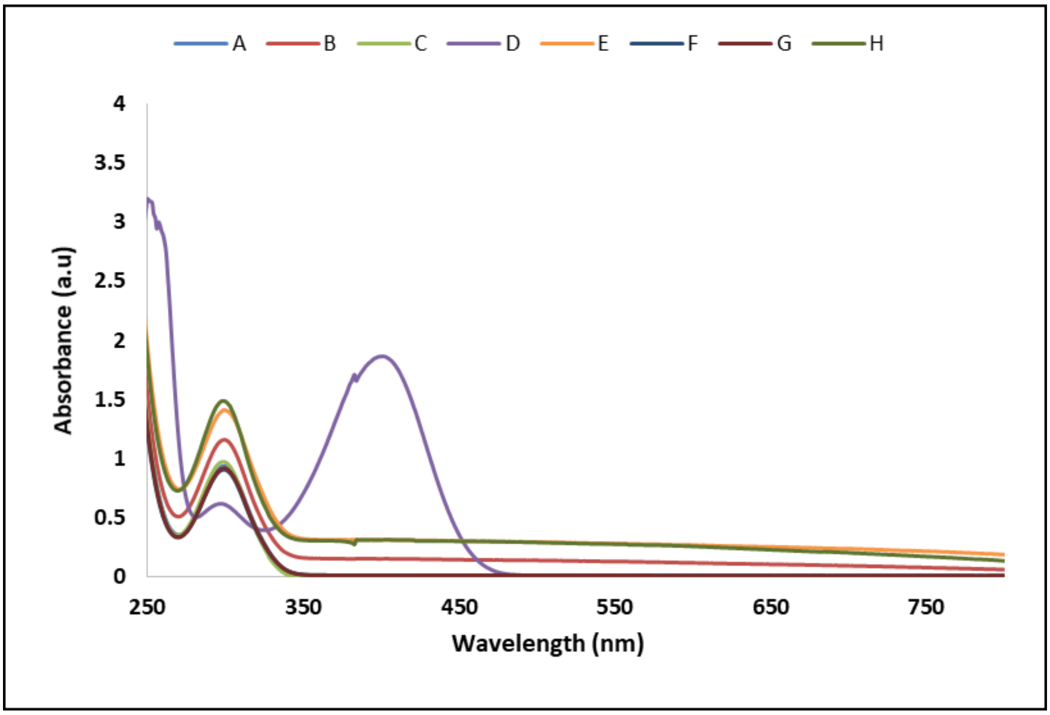

Figure S10. UV-Visible absorption spectra Optimization of amount of n-HA/Si-PA-SC@Ag and $NaBH_4$ in the reduction of 4-nitrophenol in H_2O (5 mL), at room temperature. A (10 mmol of $NaBH_4$, 20 mg of catalyst), B (7.5 mmol of $NaBH_4$, 20 mg of catalyst), C (5 mmol of $NaBH_4$, 20 mg of catalyst), D (2.5 mmol of $NaBH_4$, 20 mg of catalyst), E (8.5 mmol of $NaBH_4$, 20 mg of catalyst), F (7.5 mmol of $NaBH_4$, 30 mg of catalyst), G (10 mmol of $NaBH_4$, 30 mg of catalyst), H(7.5 mmol of $NaBH_4$, 40 mg of catalyst).

Table S3. Optimization of the reaction condition of reduction of *p*-NP.[a]

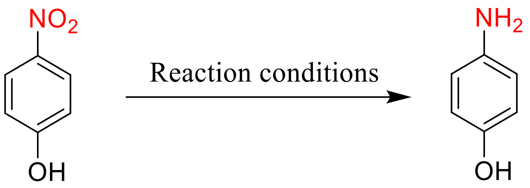

Entry	**Catalyst** (mg)	**$NaBH_4$** (mmol)	**H_2O** (mL)	**Conversion** [b] (%)	**Time** (min:sec)
1	Ag@n-HA@SiO_2-PA-SC (20)	10	5	100	5:00
2	Ag@n-HA@SiO_2-PA-SC (20)	7.5	5	100	10:00
3	Ag@n-HA@SiO_2-PA-SC (20)	5	5	100	35:00
4	Ag@n-HA@SiO_2-PA-SC (20)	2.5	5	40	50:00
5	Ag@n-HA@SiO_2-PA-SC (20)	8.5	5	100	8:00
6	Ag@n-HA@SiO_2-PA-SC (30)	7.5	5	100	7:00
7	Ag@n-HA@SiO_2-PA-SC (30)	10	5	100	4:30
8	Ag@n-HA@SiO_2-PA-SC (40)	7.5	5	100	6:00
9	Ag@n-HA@SiO_2-PA-SC (30)	7.5	2.5	100	5:00
10	Ag@n-HA@SiO_2-PA-SC (30)	-	2.5	35	240:00
11	-	7.5	2.5	20	240:00
12	n-HA (30)	7.5	2.5	Trace	5:00
13	n-HA@SiO_2 (30)	7.5	2.5	Trace	5:00
14	n-HA@SiO_2-Cl (30)	7.5	2.5	Trace	5:00
15	n-HA@SiO_2-PA (30)	7.5	2.5	Trace	5:00
16	n-HA@SiO_2-PA-SC (30)	7.5	2.5	10	5:00
17	$AgNO_3$ [c]	7.5	2.5	70	240

[a] Reaction condition: *p*-NP (0.5 mmol) under different conditions in H_2O at r.t.

[b] Isolated yields.

[c] A solution $AgNO_3$ (0.28 ppm) was prepared in which the percentage of silver was equal to the amount of silver in the catalyst structure.

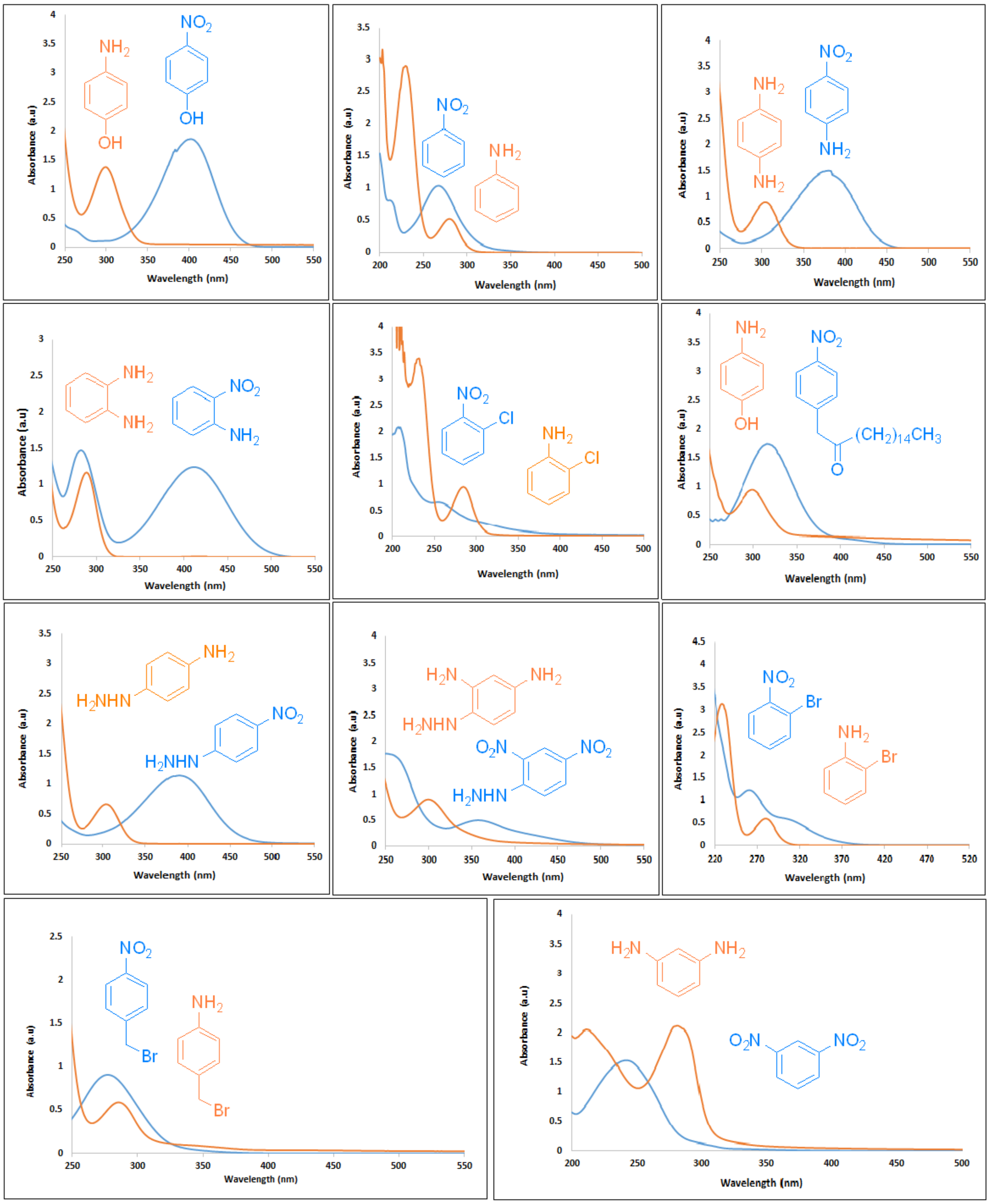

Figure S11. UV-Vis spectra of reduction reaction of NAs to AAs over $NaBH_4$ (7.5 mmol) and n-HA/Si-PA-SC@Ag (30 mg) in water (2.5 mL) at r.t.

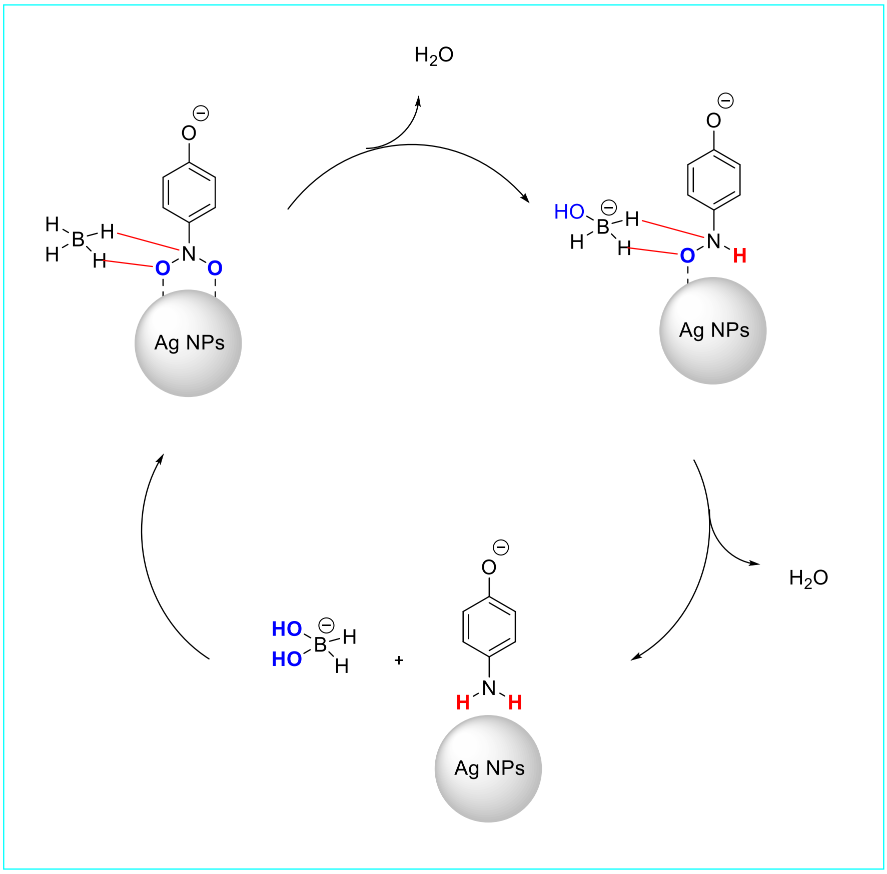

Figure S12. The recommended mechanism for the reduction of *p*-NP by $NaBH_4$ and n-HA/Si-PA-SC@Ag [7]

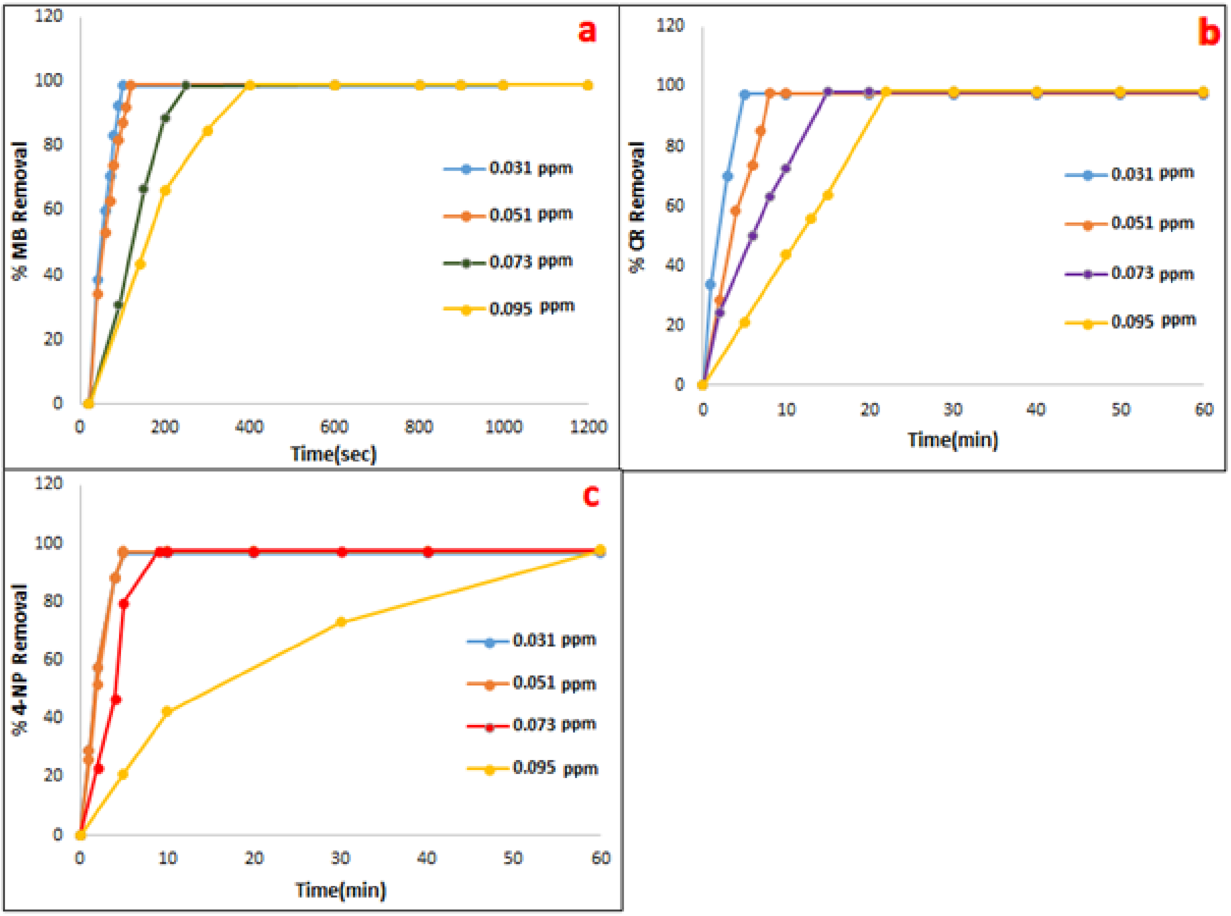

Figure S13. Effect of initial concentrations on (a) MB, (b) CR and (c) *p*-NP reduction

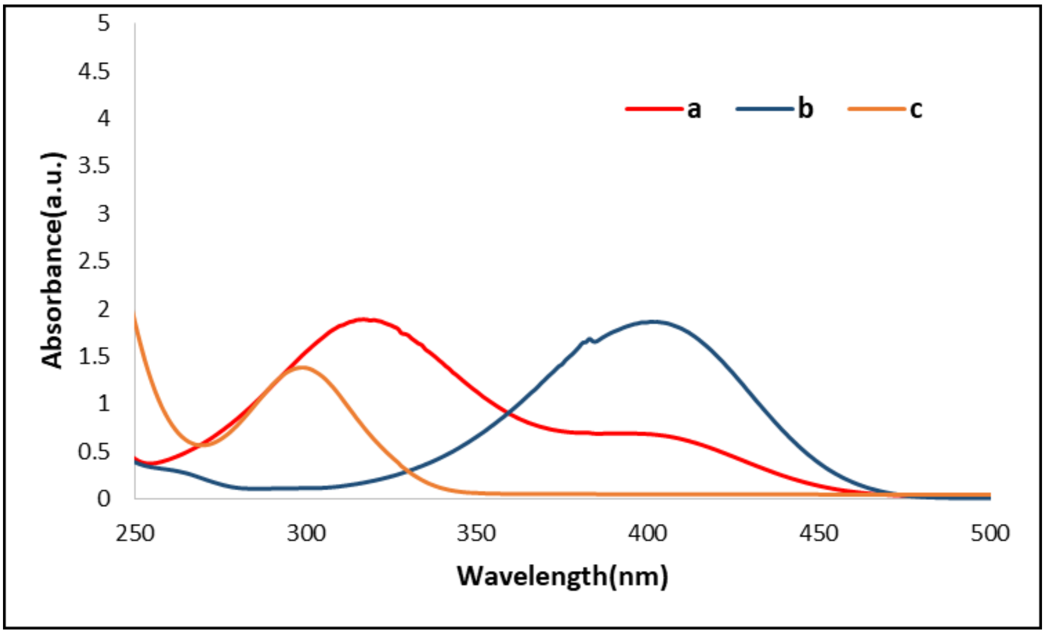

Figure S14. UV-Visible spectra of the reduction of *p*-NP (0.5 mmol) over n-HA/Si-PA-SC@Ag (30 mg) without $NaBH_4$ in 5min (a) and with $NaBH_4$ in 0 min (b) and with $NaBH_4$ in 5 min (c)

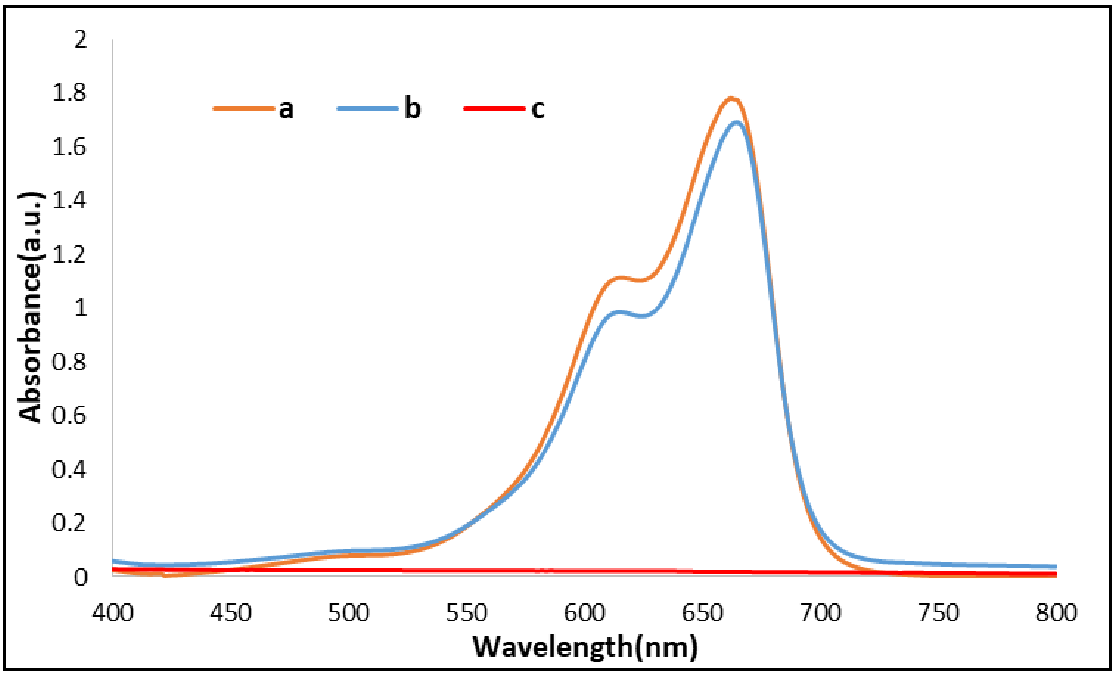

Figure S15. UV-Visible spectra of the reduction of MB (0.5 mmol) over n-HA/Si-PA-SC@Ag (300 mg) without $NaBH_4$ in 2 min (a) and with $NaBH_4$ in 20 second (b) and with $NaBH_4$ in 2 min (c)

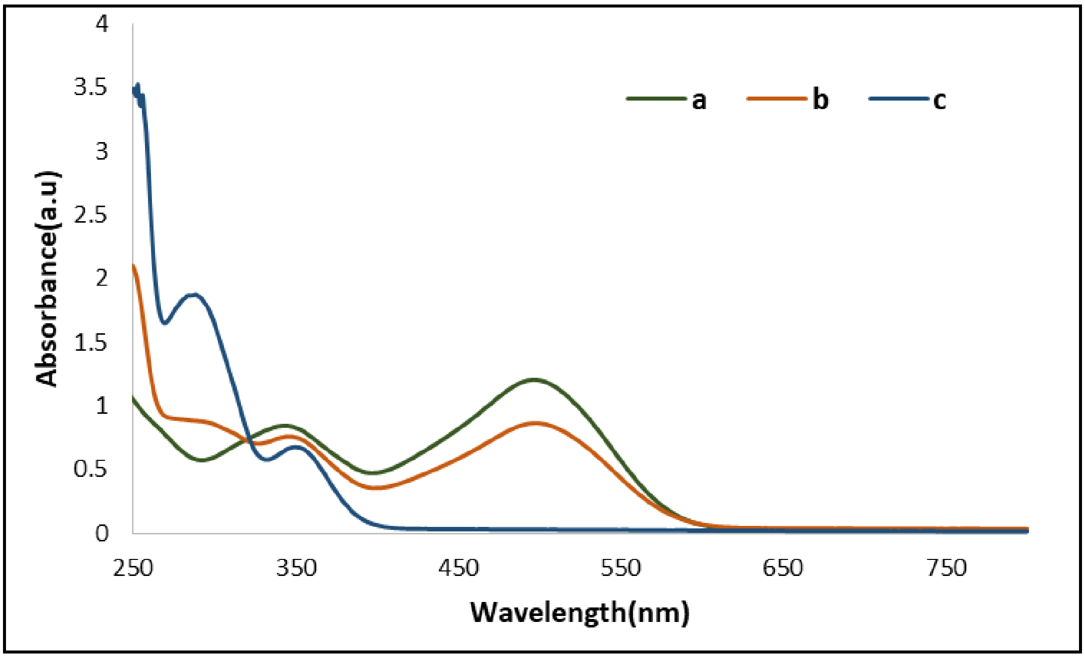

Figure S16. UV-Visible spectra of the reduction of CR (0.5 mmol) over n-HA/Si-PA-SC@Ag (300 mg) without $NaBH_4$ in 8 min (a) and with $NaBH_4$ in 1 min (b) and with $NaBH_4$ in 8 min (c)

Table S4. The catalytic activity comparison of this work with previous literatures of catalytic reduction of *p*-NP to *p*-AP (1-7), reduction of methylene blue (8-11), reduction of congo red (12-14).

Entry	Catalyst	Conditions Solvent/ Temperature (˚C)	Time (min)	Yield (%)	Ref.
1	IT-MHAP-Ag (60 mg)	H_2O/ Reflax	35	98	[8]
2	Fe_3O_4@nSiO_2@mSiO_2/Pr-Imi-NH_2-Ag (20 mg)	H_2O/ 95 ˚C	45	90	[9]
3	Pd/PVP-PS (60 mg)	H_2O/ r.t.	60	100	[10]
4	ZnS (10 mg)	H_2O/ r.t.	35	54.31	[11]
5	ZnS-RGO (10 mg)	H_2O/ under simulated solar light illumination	70	87	[12]
6	Pd NPs/RGO (6 mg)	EtOH:H_2O (v/v = 1:2)/ 50 ˚C	90	97	[13]
**7**	**n-HA/Si-PA-SC@Ag (30 mg)**	**H_2O/ r.t.**	**5**	**100**	**This work**
8	ZBD@Ag (0.2 mg/mL)	H_2O/ r.t.	6	99	[14]
9	AgNPs (0.2 mL)	H_2O/ r.t.	14	98	[15]
10	1.0Au/MMZ (1 mg)	H_2O/ r.t.	5	100	[16]
**11**	**n-HA/Si-PA-SC@Ag (300 mg)**	**H_2O/ r.t.**	**2**	**100**	**This work**
12	PPy/AgNPs -coated SSM	H_2O/ r.t.	10	86.05	[17]
13	Cd-p(NIPAM-AAc)/p(AAc) H-MIG (0.5 mL)	H_2O/r.t.	67	89.9	[18]
**14**	**n-HA/Si-PA-SC@Ag (300 mg)**	**H_2O/ r.t.**	**8**	**100**	**This work**

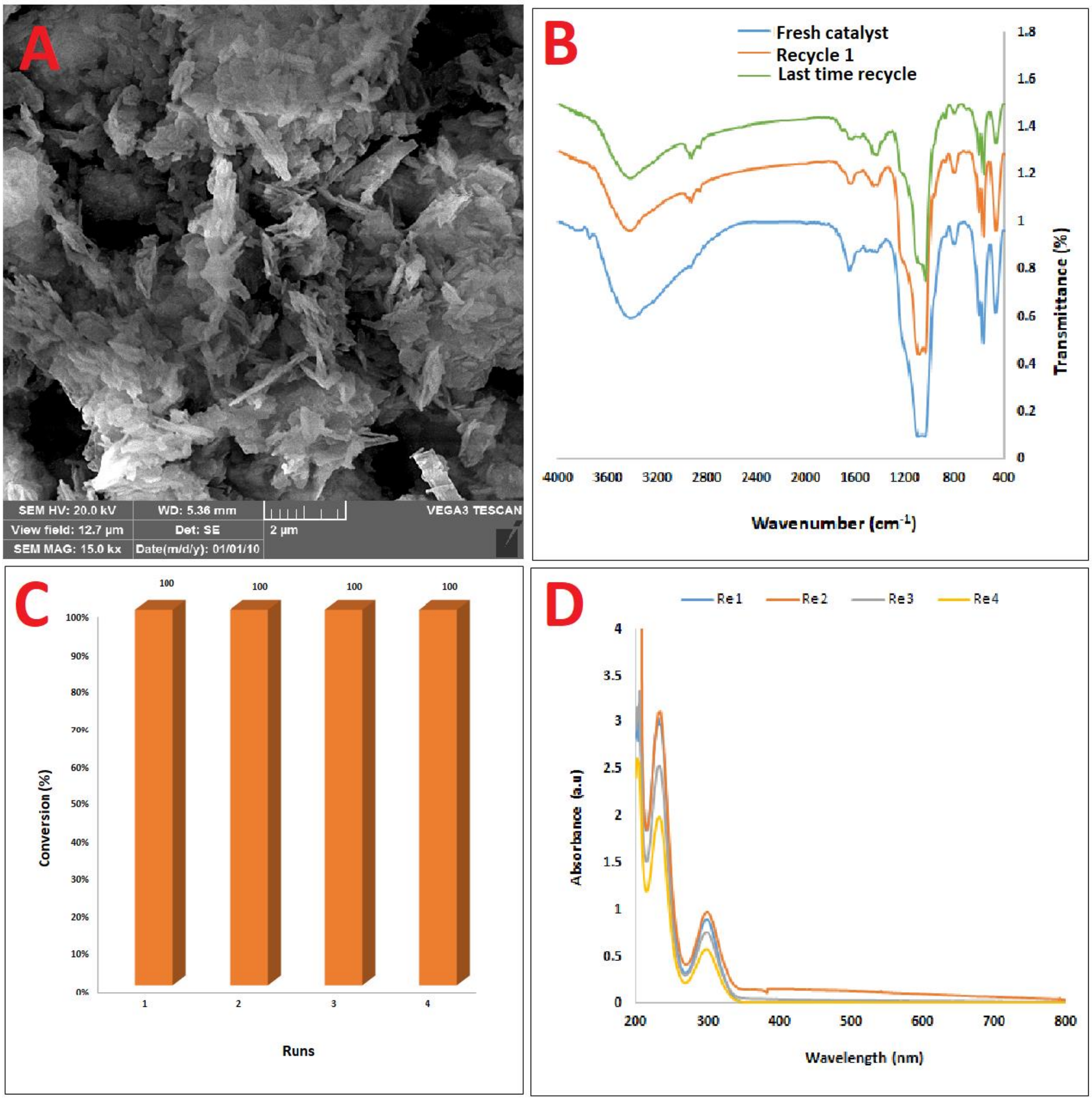

Figure S17. The SEM image (A) and FTIR spectra (B) of the recycled n-HA/Si-PA-SC@Ag and reusability runs (C) and UV-Visible spectra of the reduction of *p*-NP of over recycled n-HA/Si-PA-SC@Ag.

Characterization data

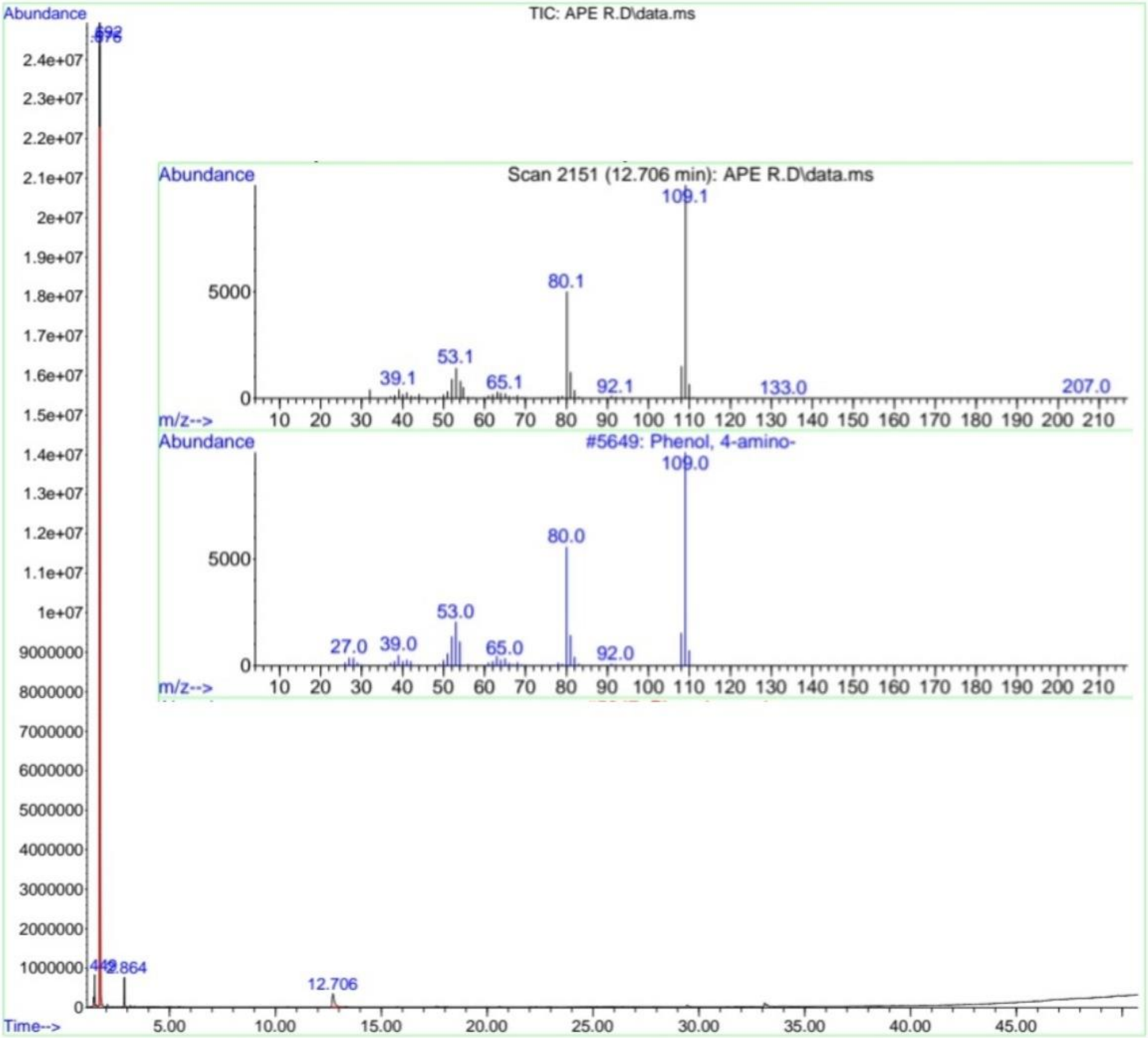


```
Data File: E:\bita\APE R.D
Sample   :

Peak Number:  5     at     12.706 min  Area:   2589209  Area % 3.67

                                             Ref\#     CAS\#      Qual
---------------------------------------------------------------------
    C:\Database\NIST14.L
     1 Phenol, 4-amino-                          5649 000123-30-8   94
```

Figure S18. The GC-MS results of 4-Aminophenol product after extracting the reaction mixture with ethyl acetate.

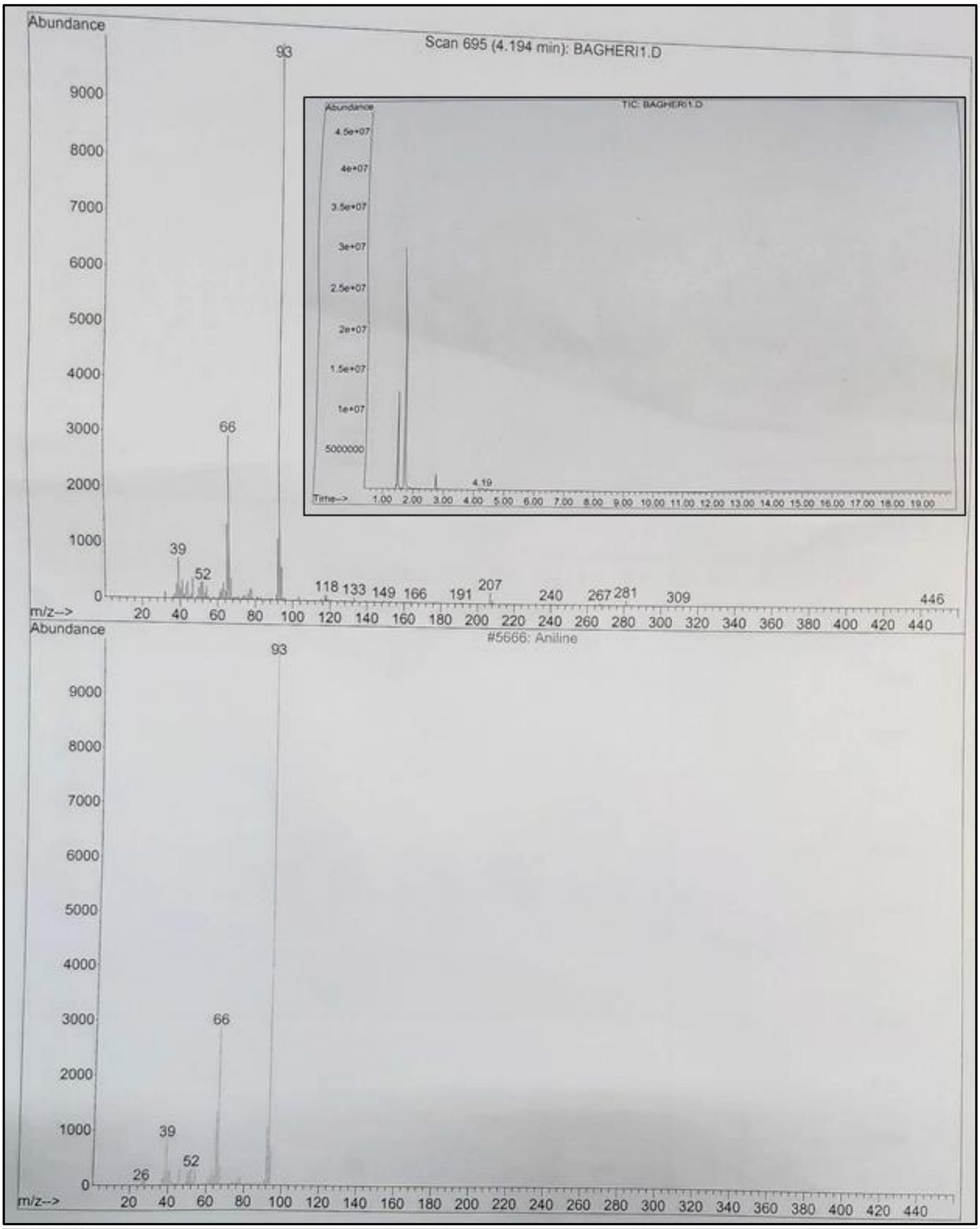

peak #	R.T. min	first scan	max scan	last scan	PK TY	peak height	corr. area	corr. % max.	% of total
1	4.188	665	694	825	M3	254043	41265048	100.00%	100.000%

Sum of corrected areas: 41265048

Figure S19. The GC-MS results of Aniline product after extracting the reaction mixture with ethyl acetate

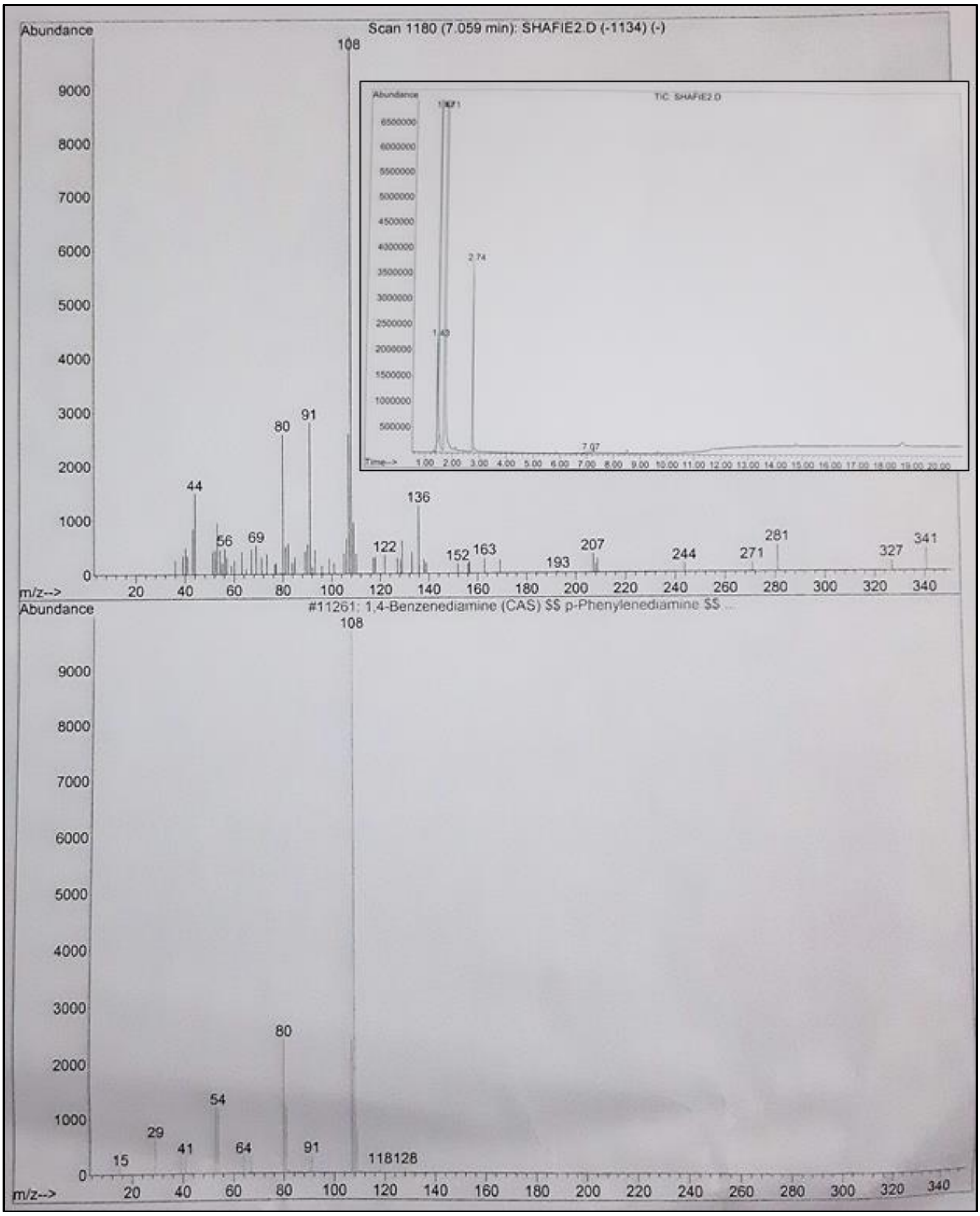

Figure S20. The GC-MS results of 4-Aminoaniline product after extracting the reaction mixture with ethyl acetate

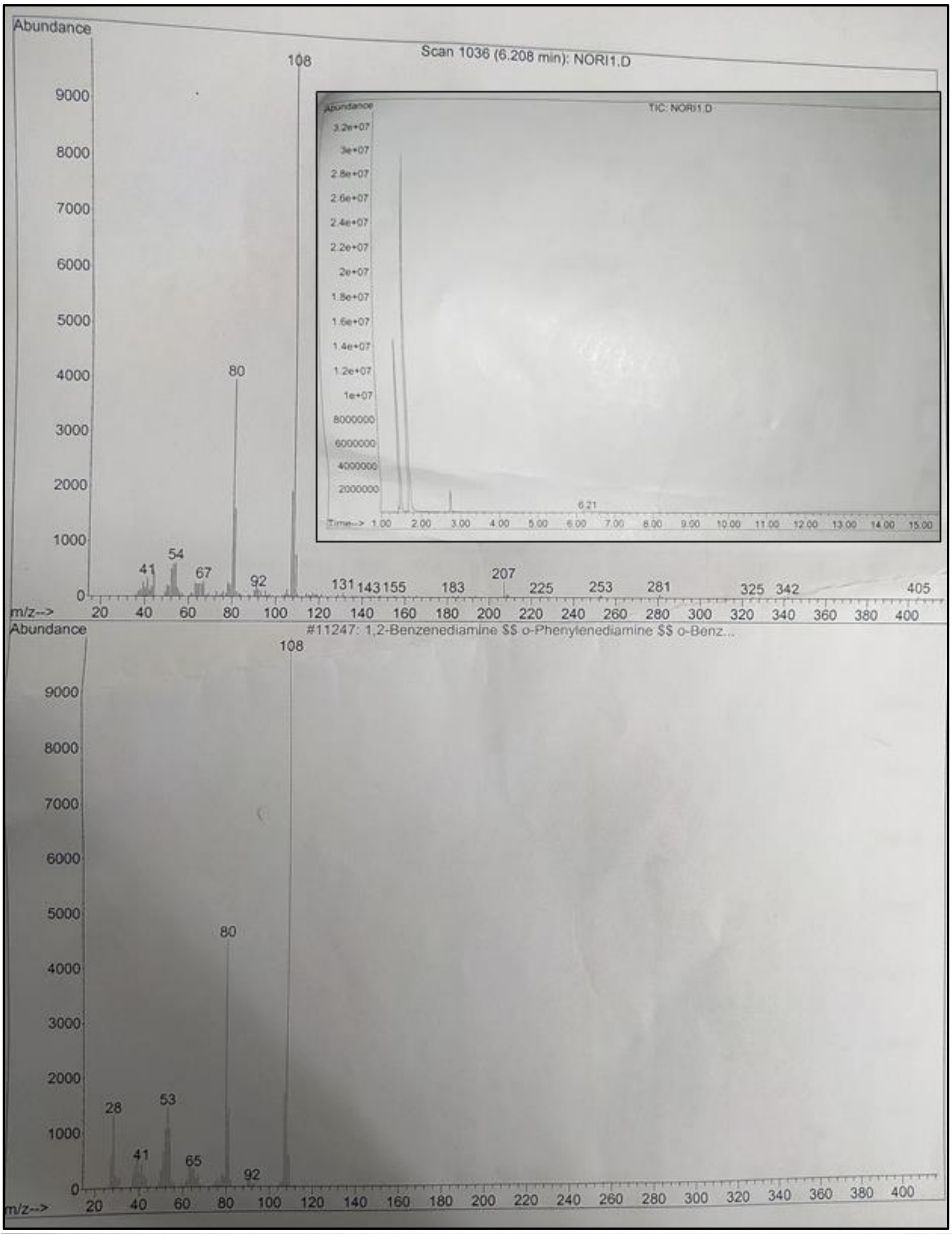

peak #	R.T. min	first scan	max scan	last scan	PK TY	peak height	corr. area	corr. % max.	% of total
1	6.214	988	1037	1126	M4	179260	35662686	100.00%	100.000%

Sum of corrected areas: 35662686

Figure S21. The GC-MS results of 2-Aminoaniline product after extracting the reaction mixture with ethyl acetate

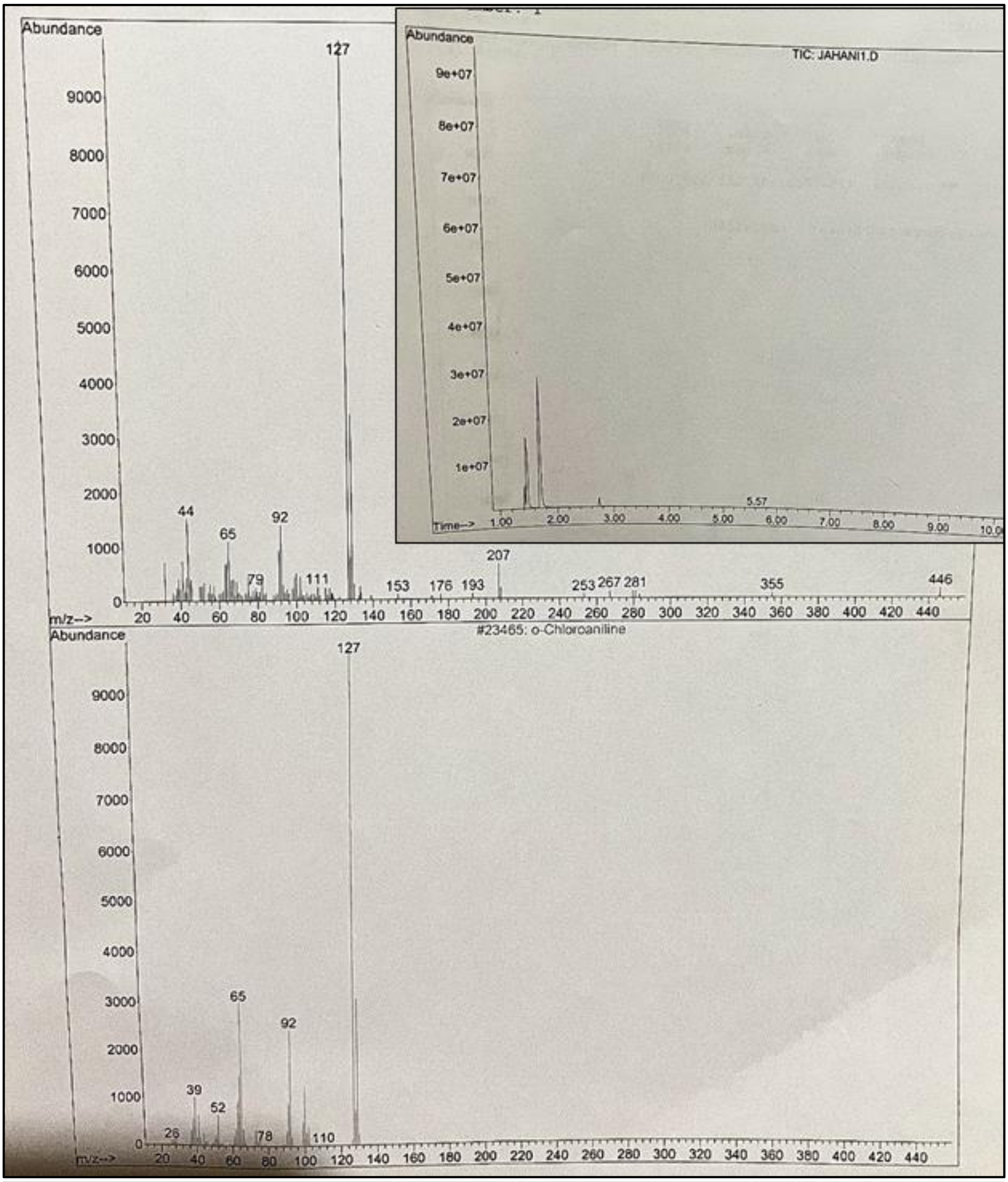

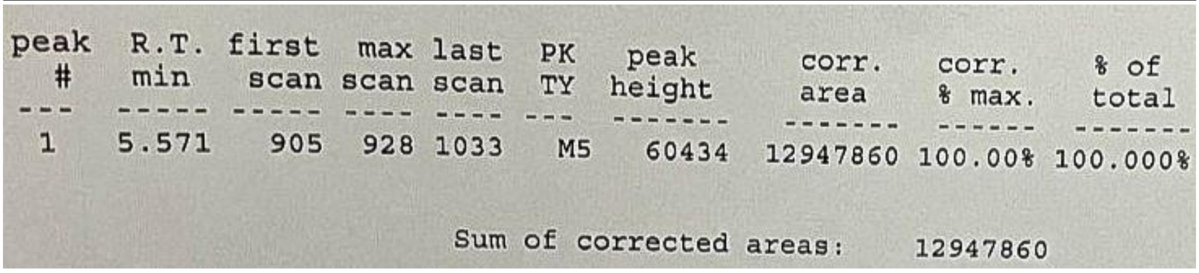

peak #	R.T. min	first scan	max scan	last scan	PK TY	peak height	corr. area	corr. % max.	% of total
1	5.571	905	928	1033	M5	60434	12947860	100.00%	100.000%

Sum of corrected areas: 12947860

Figure S22. The GC-MS results of 2-Chloroaniline product after extracting the reaction mixture with ethyl acetate.

References

1 Muralithran, G. & Ramesh, S. The effects of sintering temperature on the properties of hydroxyapatite. *Ceram. Int.* **26**, 221-230, doi:https://doi.org/10.1016/S0272-8842(99)00046-2 (2000).

2 Ta, T. K. H. *et al.* Synthesis and surface functionalization of Fe_3O_4-SiO_2 core-shell nanoparticles with 3-glycidoxypropyltrimethoxysilane and 1,1′-carbonyldiimidazole for bio-applications. *Colloids Surf. A Physicochem. Eng. Asp.* **504**, 376-383, doi:https://doi.org/10.1016/j.colsurfa.2016.05.008 (2016).

3 Mondal, J. *et al.* One-pot thioetherification of aryl halides with thiourea and benzyl bromide in water catalyzed by Cu-grafted furfural imine-functionalized mesoporous SBA-15. *Chem. Commun.* **48**, 8000-8002, doi:https://doi.org/10.1039/C2CC32676K (2012).

4 Manafi, S. A. *et al.* Synthesis of nano-hydroxyapatite under a sonochemical/hydrothermal condition. *Biomed Mater* **3**, 025002-025009, doi:https://doi.org/10.1088/1748-6041/3/2/025002 (2008).

5 Farhadi, S., Ajerloo, B. & Mohammadi, A. Low-cost and eco-friendly phyto-synthesis of Silver nanoparticles by using grapes fruit extract and study of antibacterial and catalytic effects. *Int. J. Nano Dimens.* **8**, 49-60, doi:https://doi.org/10.22034/ijnd.2017.24376 (2017).

6 Yuan, P. *et al.* Functionalization of halloysite clay nanotubes by grafting with γ-aminopropyltriethoxysilane. *J. Phys. Chem. C* **112**, 15742-15751, doi:https://doi.org/10.1021/jp805657t (2008).

7 Ismail, M. *et al.* Catalytic reduction of picric acid, nitrophenols and organic azo dyes via green synthesized plant supported Ag nanoparticles. *J. Mol. Liq.* **268**, 87-101, doi:https://doi.org/10.1016/j.molliq.2018.07.030 (2018).

8 Pashaei, M. & Mehdipour, E. Silver nanoparticles supported on ioni*f* tagged magnetic hydroxyapatite as a highly efficient and reusable nanocatalyst for hydrogenation of nitroarenes in water. *Appl. Organomet. Chem.* **32**, 4226-4236, doi:https://doi.org/10.1002/aoc.4226 (2018).

9 Jafari Nasab, M. & Kiasat, A. R. Multifunctional Fe_3O_4@nSiO_2@mSiO_2/Pr-Imi-NH_2·Ag core–shell microspheres as highly efficient catalysts in the aqueous reduction of nitroarenes: improved catalytic activity and facile catalyst recovery. *RSC Adv.* **6**, 41871-41877, doi:https://doi.org/10.1039/C6RA00120C (2016).

10 Lakshminarayana, B., Ashok Kumar, K. V., Selvaraj, M., Satyanarayana, G. & Ch, S. PVP-PS supported ultra-small Pd nanoparticles for the room temperature reduction of 4-nitrophenol. *J. Environ. Chem. Eng.* **8**, 103899-103917, doi:https://doi.org/10.1016/j.jece.2020.103899 (2020).

11 Ayodhya, D. & Veerabhadram, G. Stable and efficient graphitic carbon nitride nanosheet–supported ZnS composite catalysts toward competent catalytic performance for the reduction of 4-nitrophenol using NaBH4. *Mater. Today Sustain.* **5**, 100015, doi:https://doi.org/10.1016/j.mtsust.2019.100015 (2019).

12 Ibrahim, S., Chakrabarty, S., Ghosh, S. & Pal, T. Reduced Graphene Oxide - Zinc Sulfide Composite for Solar Light Responsive Photo Current Generation and Photocatalytic 4-Nitrophenol Reduction. *ChemistrySelect* **2**, 537-545, doi:https://doi.org/10.1002/slct.201601999 (2017).

13 Nasrollahzadeh, M., Sajadi, S. M., Rostami-Vartooni, A., Alizadeh, M. & Bagherzadeh, M. Green synthesis of the Pd nanoparticles supported on reduced graphene oxide using barberry fruit extract and its application as a recyclable and heterogeneous catalyst for the reduction of nitroarenes. *J. Colloid Interface Sci.* **466**, 360-368, doi:10.1016/j.jcis.2015.12.036 (2016).

14 Lajevardi, A., Tavakkoli Yaraki, M., Masjedi, A., Nouri, A. & Hossaini Sadr, M. Green synthesis of MOF@Ag nanocomposites for catalytic reduction of methylene blue. *J. Mol. Liq.* **276**, 371-378, doi:https://doi.org/10.1016/j.molliq.2018.12.002 (2019).

15 Mehata, M. S. Green route synthesis of silver nanoparticles using plants/ginger extracts with enhanced surface plasmon resonance and degradation of textile dye. *Mater. Sci. Eng. B* **273**, 115418-115426, doi:https://doi.org/10.1016/j.mseb.2021.115418 (2021).

16 Aslam, S., Subhan, F., Yan, Z., Yaseen, M. & Shujahat, M. H. Fabrication of gold nanoparticles within hierarchically ZSM-5-based micro-/mesostructures (MMZ) with enhanced stability for catalytic reduction of p-nitrophenol and methylene blue. *Sep. Purif. Technol.* **254**, 117645-117654, doi:https://doi.org/10.1016/j.seppur.2020.117645 (2021).

17 Yihan, S., Mingming, L. & Guo, Z. Ag nanoparticles loading of polypyrrole-coated superwetting mesh for on-demand separation of oil-water mixtures and catalytic reduction of aromatic dyes. *J. Colloid Interface Sci.* **527**, 187-194, doi:https://doi.org/10.1016/j.jcis.2018.05.048 (2018).

18 Ullah, B., Khan, S. R., Ali, S., Jamil, S. & Saeed Ashraf Janjua, M. R. 4-Nitrophenol imprinted core-shell poly(N-isopropylacrylamide-acrylic acid)/poly(acrylic acid) microgels loaded with cadmium nanoparticles: A novel catalyst. *Mater. Chem. Phys.* **260**, 124156-124170, doi:https://doi.org/10.1016/j.matchemphys.2020.124156 (2021).
